# Water‐Immersed GaP Huygens’ Meta‐Optics for Visible Structured Light Generation

**DOI:** 10.1002/advs.202510467

**Published:** 2025-08-11

**Authors:** Jia‐Hua Lee, Hsing‐Yi Wang, Pei Ying Ho, Yu Chia Chung, Ruei‐Tzu Duh, Cheng‐Ching Chiang, Ray‐Hua Horng, Yao‐Wei Huang, Ming Lun Tseng

**Affiliations:** ^1^ Institute of Electronics College of Electrical and Computer Engineering National Yang Ming Chiao Tung University Hsinchu 300010 Taiwan; ^2^ Department of Photonics College of Electrical and Computer Engineering National Yang Ming Chiao Tung University Hsinchu 300010 Taiwan

**Keywords:** GaP, Huygens’ metasurface, Kerker effect, structured light, underwater photonics

## Abstract

Underwater photonics is essential for imaging and analysis in biomedical technologies, as well as for communication and autonomous vehicle networks in the Internet of Underwater Things (IoUT). Structured light enables spatial control of beams, offering new opportunities for underwater sensing, imaging, and signal transmission. However, compact and robust water‐immersed photonic devices remain challenging to realize. Among various approaches, dielectric metasurfaces are particularly promising for underwater optics due to their flat form factor and subwavelength phase control. Nevertheless, current platforms suffer from instability in liquids, incomplete phase modulation, or strong sensitivity to fabrication imperfections. Here, ultrathin water‐immersed metasurfaces composed of gallium phosphide (GaP) Huygens’ integrated resonance units (HIRUs) operating in the visible regime are demonstrated. By leveraging the high refractive index and low optical loss of GaP, and integrating both forward and inverse design strategies, metasurfaces with planar geometry (≈1/5 of the operating wavelength), enhanced fabrication tolerance, and stable in‐liquid operation are realized. These metasurfaces generate various structured light beams in water, including abruptly autofocusing (AAF) beams, Bessel beams, optical vortices, and Gaussian focusing beams. This work establishes a scalable and reliable platform for underwater nanophotonics and emerging IoUT‐related applications.

## Graphical abstract

Ultrathin GaP Huygens’ metasurfaces operating in the visible regime generate structured light beams underwater, including focusing Gaussian beams, abruptly autofocusing beams, Bessel beams, and optical vortices. The platform offers enhanced fabrication tolerance and mechanical stability, establishing a robust route toward compact underwater nanophotonics for biomedical and IoUT applications.

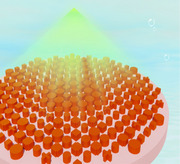

## 1 Introduction

Efficient manipulation of light in liquid environments is critical for a wide range of technologies, including biomedical processes,^[^ ^1^, ^2^, ^3^, ^4^ ^]^ and the emerging Internet of Underwater Things (IoUT).^[^ ^5^, ^6^, ^7^ ^]^ In biomedical applications, aqueous media form the natural environment for most biological specimens, making in‐liquid photonics indispensable for in vivo biomedical imaging^[^ ^1^, ^3^ ^]^ and molecular spectroscopic analysis.^[^ ^4^ ^]^ In IoUT, underwater photonic technologies are essential for enabling high‐capacity sensing, underwater wireless optical communication (UWOC),^[^ ^5^, ^7^ ^]^ and autonomous navigation.^[^ ^6^, ^8^ ^]^

Among recent advances, structured light^[^ ^9^ ^]^ has emerged as a powerful tool for enhancing optical performance in liquid environments. In particular, Bessel beams,^[^ ^10^ ^]^ characterized by non‐diffracting propagation, self‐healing behavior, and extended depth of focus due to their propagation‐invariant nature,^[^ ^11^, ^12^ ^]^ have been widely explored for underwater biomedical imaging,^[^ ^13^, ^14^, ^15^ ^]^ and particle manipulation.^[^ ^16^, ^17^ ^]^ Similarly, Airy beams^[^ ^18^ ^]^ and abruptly autofocusing (AAF) beams,^[^ ^19^, ^20^, ^21^ ^]^ also referred to as ring‐Airy beams, exhibit self‐accelerating curved trajectories and fall within the class of propagation‐invariant structured light. AAF beams are particularly notable for their nonlinear axial intensity evolution. They can be generated by imposing a circularly symmetric cubic phase profile onto the wavefront, either through a spatial light modulator or directly via a metasurface design. Unlike conventional focusing beams, the optical energy in AAF beams remains distributed along the propagation path and then abruptly concentrates at a predefined focal distance. This sharp axial localization produces high contrast at the focal point and makes AAF beams especially useful for remote energy delivery, nonlinear excitation, and high‐precision photonic applications and have been used for compact imaging systems, biospecimen stimulation^[^ ^22^ ^]^ and high‐fidelity data transmission in aquatic communication networks.^[^ ^23^ ^]^ In addition, optical vortices, which carry orbital angular momentum (OAM), provide additional information channels and enable advanced applications such as multiplexed UWOC,^[^ ^24^, ^25^ ^]^ particle rotation,^[^ ^26^ ^]^ and contrast‐enhanced imaging.^[^ ^27^ ^]^ However, conventional generation of structured light typically relies on bulky and delicate optical assemblies, including axicons, spatial light modulators, and diffractive optical elements (DOE), which limit system miniaturization, mechanical stability, and integration into space‐restricted environments typically encountered in underwater photonic platforms. A compact, robust photonic platform capable of generating structured light directly within liquid media is therefore highly desirable. The advancement of compact underwater optical components for structured light generation is therefore key to driving innovation and enabling system‐level miniaturization.

Metasurfaces^[^ ^28^, ^29^, ^30^ ^]^ have emerged as a powerful platform for underwater photonics. With nanoscale geometry and exceptional design flexibility, metasurfaces have enabled breakthroughs in wavefront control,^[^ ^31^, ^32^, ^33^, ^34^, ^35^ ^]^ quantum optics,^[^ ^36^, ^37^ ^]^ chiral optics,^[^ ^38^, ^39^, ^40^ ^]^ display and edge detection,^[^ ^41^, ^42^ ^]^ biosensing,^[^ ^43^, ^44^, ^45^, ^46^, ^47^, ^48^ ^]^ biomedical imaging,^[^ ^49^, ^50^ ^]^ and light generation^[^ ^51^, ^52^, ^53^ ^]^  by precisely arranging subwavelength resonant elements (meta‐atoms). Due to their ability to tailor the amplitude, phase, and polarization response at will,^[^ ^54^, ^55^, ^56^ ^]^ metasurfaces have also been widely used to generate structured light under air conditions for applications ranging from biophotonics to high‐capacity communication systems.^[^ ^22^, ^57^, ^58^, ^59^, ^60^ ^]^ Several studies have extended metasurface functionalities into aqueous environments.^[^ ^23^, ^61^, ^62^, ^63^, ^64^ ^]^ In many cases, these metasurfaces are designed to operate in air and direct light into a liquid‐filled container.^[^ ^23^, ^61^, ^62^ ^]^ While this configuration enables a range of novel applications, it imposes significant limitations on key design parameters, such as the working distance. A more versatile approach involves the development of metasurfaces that operate directly within liquid media, known as front‐immersion metasurfaces. Achieving full‐wave control in this context requires sufficient dielectric contrast between the resonant unit cells and the surrounding medium. By employing high‐aspect‐ratio unit cell designs (typically with aspect ratios between 5 and 10), researchers have successfully demonstrated immersion metalenses.^[^ ^63^, ^64^, ^65^ ^]^ The working principle relies on forming Fabry‐Pérot‐like, low‐quality‐factor waveguiding modes inside the nanopillar structures,^[^ ^66^, ^67^ ^]^ where phase modulation can be gradually accumulated along the propagation through the nanopillars. Pioneering demonstrations using dielectric materials (e.g., GaN) have investigated the feasibility of water‐immersed metasurfaces based on high‐aspect‐ratio designs. However, for underwater operation, the higher refractive index of water significantly reduces the dielectric contrast between the resonant unit cells and the surrounding medium compared to in‐air conditions. This weakened contrast leads to reduced optical confinement and resonance strength, making it difficult to achieve full 2π phase shifts^[^ ^64^ ^]^ and potentially limiting device performance in practical scenarios. The fabrication of high‐aspect‐ratio nanopillars typically demands highly precise dry etching conditions, which substantially increases experimental complexity. In addition, directly immersing such high‐aspect‐ratio nanopillars in a solvent presents challenges, including stability issues in flowing environments and contamination from dust and particles floating around the devices. The high aspect ratio and nanoscale feature size of the unit cells also complicate the application of protective coatings using standard semiconductor packaging techniques such as conformal coating or thin‐film deposition.^[^ ^68^ ^]^ This limitation hinders the stabilization of these devices through well‐established semiconductor manufacturing processes. To circumvent these issues, a back‐immersion scheme was proposed,^[^ ^62^ ^]^ where the metasurface and the solution are separated by a transparent substrate. In this configuration, the light passing through the metasurface is manipulated before entering the solution. The substrate provides sufficient protection to the nanostructures in the metasurface. Nevertheless, the large thickness (≈mm in general) of the substrate also imposes significant restrictions on the working distance of back‐immersion metasurfaces. Plasmonic nanoparticles offer another approach for creating planar water‐immersed metasurfaces.^[^ ^69^ ^]^ While these structures show potential, their high optical losses and low transmission efficiency limit their effectiveness for transmission‐based applications. As a result, there remains a pressing need for a robust and stable device scheme capable of realizing efficient water‐immersed metasurfaces in the visible regime.

A promising strategy for realizing ultrathin water‐immersed metasurfaces in the visible spectrum is to use low‐loss compound semiconductors as the constituent material and incorporate electromagnetic multipole resonances into planar meta‐atom designs that satisfy the generalized Kerker condition (GKC).^[^ ^70^, ^71^, ^72^, ^73^, ^74^, ^75^, ^76^, ^77^ ^]^ These meta‐atoms, often called Huygens meta‐atoms due to their highly directional scattering capabilities, enable high optical transmission and full 2π phase control. Huygens’ metasurfaces have been successfully demonstrated across various wavelength ranges.^[^ ^71^, ^72^, ^73^, ^78^, ^79^, ^80^, ^81^ ^]^ Meeting the GKC typically results from near‐field interference between multiple Mie‐resonant modes with comparable strength but different parity (e.g., electric and magnetic dipoles).^[^ ^70^ ^]^ The strong multipolar resonances within the meta‐atoms enable abrupt phase modulation directly at the interface, eliminating the need for high‐aspect‐ratio geometries typically required in waveguiding‐based designs. Consequently, metasurfaces composed of Huygens’ meta‐atoms can be fabricated with very thin thicknesses, typically between one‐third to one‐fifth of the operating wavelength, and with low aspect ratios (≈1). Moreover, Huygens’ meta‐atoms generally require refractive index symmetry between their top and bottom claddings, and are therefore often encapsulated in a transparent dielectric layer (e.g., SiO_2_ or transparent polymer).^[^ ^73^, ^76^, ^77^ ^]^ This configuration not only satisfies optical requirements but also serves a practical role, as the encapsulating layer acts as a protective coating for operation in solvent environments. Compared with metasurfaces based on high‐aspect‐ratio dielectric posts, this encapsulation provides an important advantage for underwater applications by enhancing structural and operational stability in flowing liquids. In this work, we demonstrate the generation and in‐liquid operation of structured light using ultrathin metasurfaces that are fully immersed in water. These devices consist of carefully engineered Huygens’ meta‐atoms made from high‐index GaP. To ensure fabrication tolerance and robustness, we employed a variety of meta‐atom geometries. A simple yet effective particle swarm optimization (PSO) algorithm was used to streamline the design process through integration with numerical simulations. The resulting GaP meta‐atoms, with thicknesses below 100 nm, exhibit high transmittance and the necessary phase modulation across the visible range. To demonstrate their application potential, we fabricated multiple functional metasurfaces capable of generating structured light beams directly in water. The experimental results confirm that these metasurfaces can robustly operate in liquid environments, highlighting their potential for advancing water‐immersed meta‐optics in the visible regime.

## 2 Numerical Design and Theoretical Analysis

**Figure 1:**
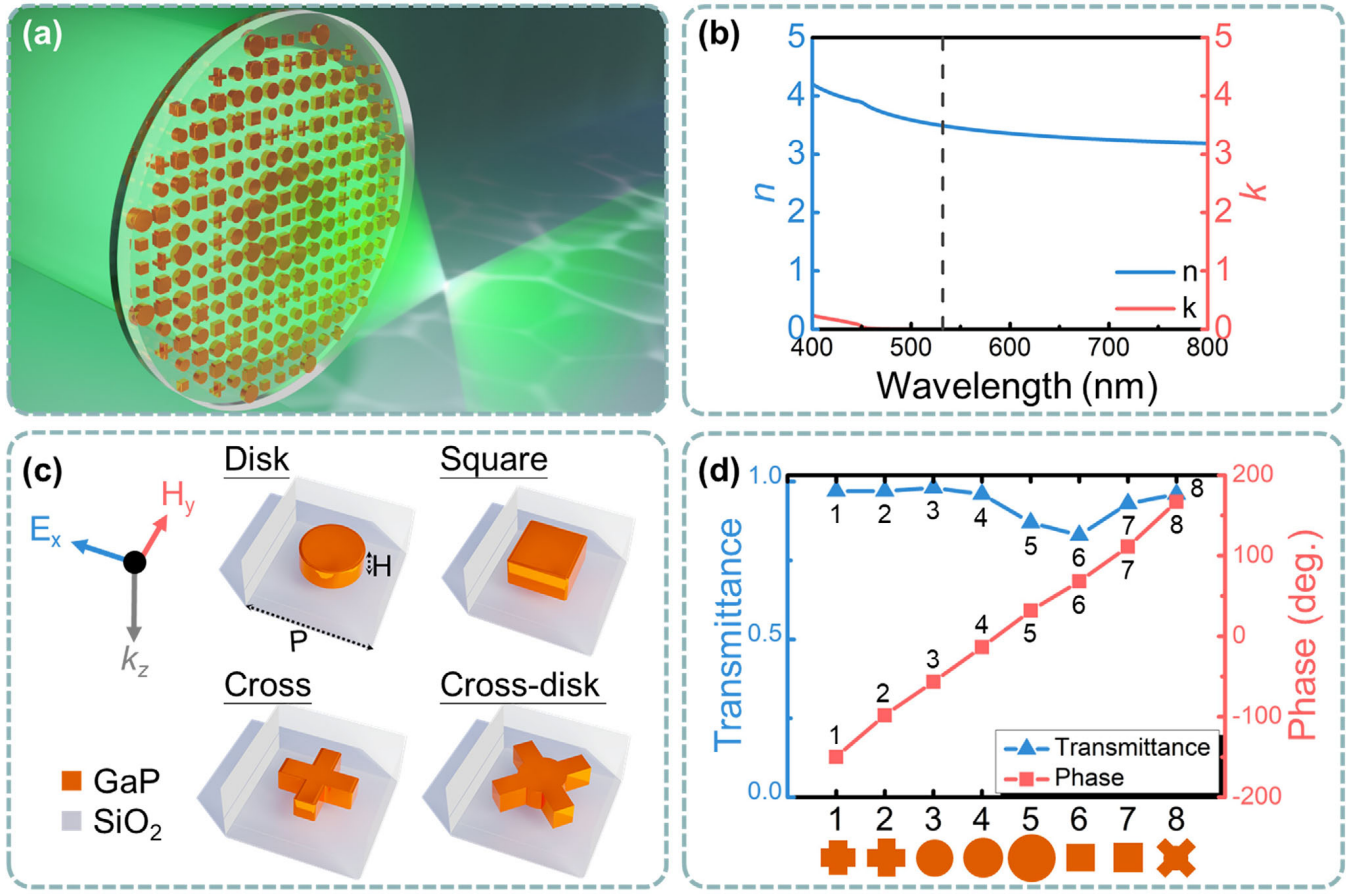
Water‐immersed Huygens's metasurfaces. a) Schematic of the metasurface comprising the planar HIRUs. b) The refractive index and extinction coefficient of GaP used in this paper. c) Schematic of the geometries used in the HIRU designs. *H*, height of the HIRUs, 85 nm. *P*, the periodic constant of the HIRUs, 320 nm. Thickness of the SiO_2_ protective layer: 300 nm. d) The transmittance and phase of the HIRUs.

**Figure 2:**
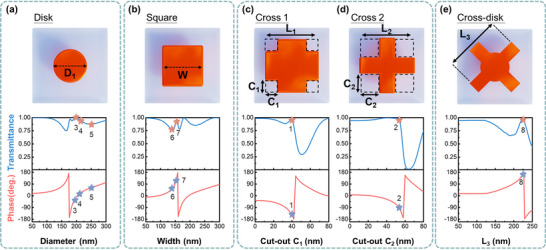
Geometry dependence of the HIRUs’ properties. The transmittance and phase of a) disk meta‐atoms versus the diameter (*D*_1_), b) square meta‐atoms versus the length (*W*), c) cross 1 versus its cut‐out sizes (labeled as *C*_1_), *L_1_* = 180 nm, d) cross 2 versus the cut‐out sizes (labeled as *C*_2_), *L_2_* = 200 nm, e) cross‐disk versus the arm length (labeled as *L*_3_).

**Figure 3:**
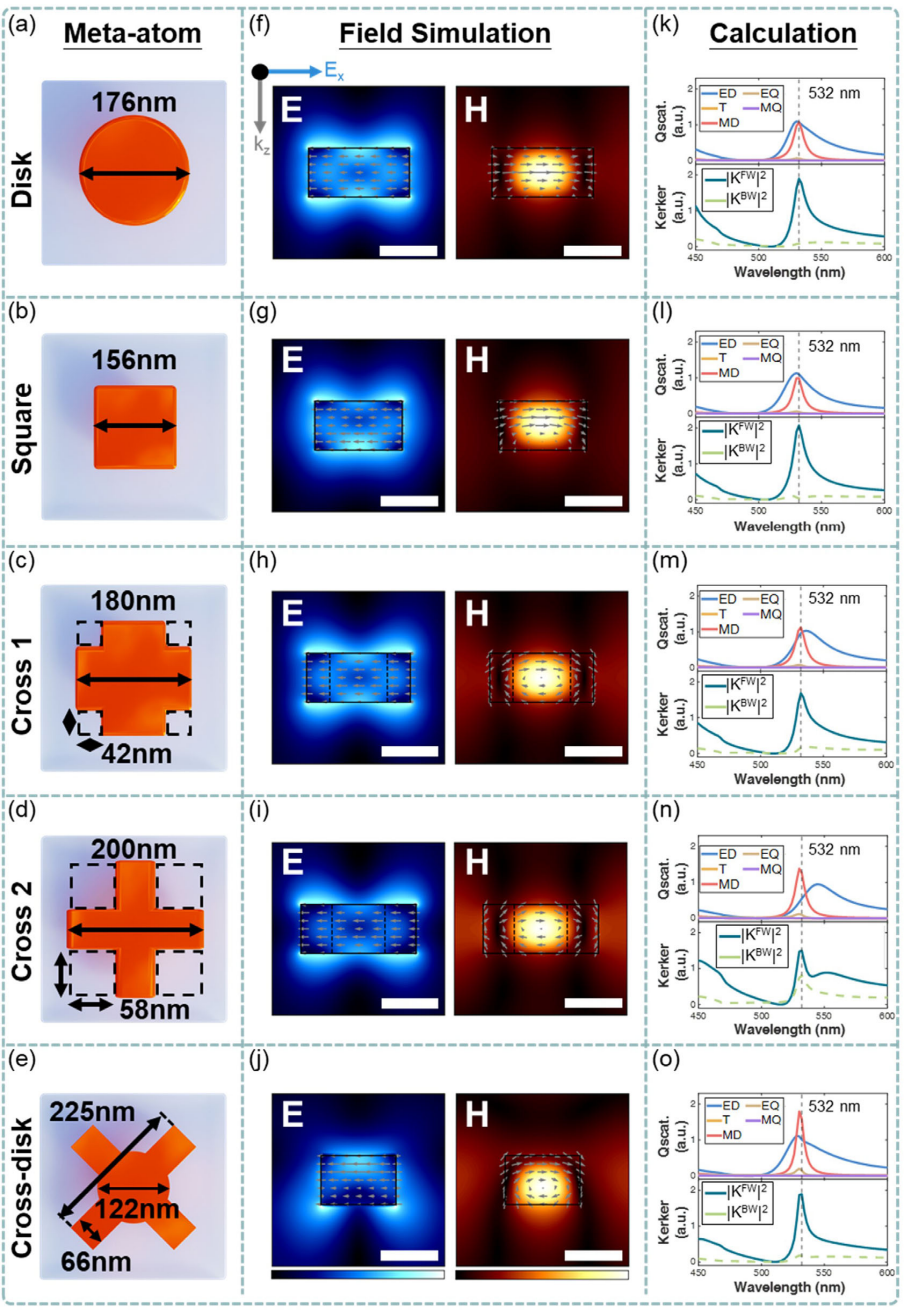
Analysis of the resonant modes and scattering properties of the HIRUs. a–e) Schematic illustrations of the meta‐atom designs analyzed. f–j) Simulated electric (left panels, blue color map) and magnetic (right panels, red color map) field distributions around the meta‐atoms under x‐polarized excitation. The meta‐atom boundaries are outlined with black dashed lines, and the gray arrows indicate the direction of the displacement current. Scale bars in (f–j): 100 nm. k–o) Calculated results showing the scattering contributions from individual multipoles (top panels) and the Kerker coefficients (bottom panels) for each meta‐atom.

There are two primary approaches to designing meta‐atoms that satisfy the generalized Kerker condition (GKC) for wavefront‐shaping metasurfaces. The first involves designing meta‐atoms that fulfill GKC under circularly polarized excitation. By incorporating geometric phase principles, local phase control can be achieved by simply rotating each meta‐atom around its central axis.^[^ ^71^, ^82^ ^]^ While efficient, this method requires additional components for polarization manipulation and filtering, making it less suitable for underwater applications where compactness is critical. The second approach relies on Huygens’ meta‐atoms with high rotational symmetry, enabling compatibility with a wide range of polarization states. This typically involves identifying a disk (or hole) structure that satisfies GKC at resonance. A full 2π phase coverage is then achieved by slightly scaling the disk diameter to generate a series of resonant elements. Although this strategy has been successfully applied in the infrared^[^ ^75^ ^]^ and GHz^[^ ^78^ ^]^ regimes, it becomes impractical at visible wavelengths, where smaller features push the limits of standard nanofabrication techniques such as electron beam lithography. This often results in fabrication inaccuracies and degraded device performance. Such imperfections introduce deviations in local phase sampling, leading to reduced operational efficiency of the visible Huygens’ metasurfaces. To overcome these limitations, we explored alternative nanoparticle geometries capable of delivering robust phase control with reduced sensitivity to fabrication imperfections, thus enabling the effective implementation of functional water‐immersed metasurfaces at visible wavelengths. **Figure** **1**a illustrates the schematic of the metasurfaces developed in this work, comprising an array of meta‐atoms embedded in a SiO_2_ layer. This configuration provides a symmetric optical background while isolating the metasurface from the surrounding aqueous environment. The device operates at a wavelength of 532 nm, which is commonly used for biological imaging and biomolecular fluorescence excitation,^[^ ^61^ ^]^ and is also suitable for underwater optical communication.^[^ ^8^, ^83^ ^]^ To ensure sufficient dielectric contrast with the SiO_2_ background, GaP was chosen as the constituent material due to its low optical loss, high refractive index at the design wavelength (Figure 1b). Previous works have also confirmed the feasibility of using GaP for realizing efficient and novel photonic devices in the visible regime.^[^ ^84^, ^85^, ^86^ ^]^ As shown in Figure 1c, we proposed a meta‐atom library, termed as Huygens’ integrated resonance units (HIRUs), that comprises four distinct geometries: disks, squares, crosses, and a hybrid structure referred to as a cross‐disk, which consists of a cross overlaid on a disk. The nanoparticle height was fixed at 85 nm, approximately one‐fifth of the effective wavelength (400 nm) in water, with the meta‐atoms arranged in a square lattice of 320 nm period. The meta‐atoms are sandwiched by a SiO_2_ substrate and a 300‐nm‐thick SiO_2_ protective layer to meet the ideal symmetric condition for exciting Huygens’ resonance.^[^ ^76^ ^]^ By tuning the geometry parameters of the HIRUs, a group of HIRUs exhibiting high transmittance (>75%), polarization insensitivity, a full 2π phase coverage, and fabrication feasibility were designed (Figure 1d). The detailed geometry parameters for the HIRUs (labeled as meta‐atoms 1–8, corresponding to phase values from −180° to 180° in ~45° steps, as also shown in **Figure** **2**), along with the design strategy and selection process, will be described in the discussion of Figure 2. The related multipole and radiation analyses will be presented in the discussion of **Figure** **3**. We note that the thickness of the SiO_2_ protective layer can be further reduced to accommodate specific application needs without significantly affecting the metasurface performance. Additionally, the reported meta‐atoms are significantly thinner than the nanofin or nanopillar unit cells commonly used in dielectric metasurfaces (typically 300–700 nm in height). The fabrication of such high‐aspect‐ratio structures typically requires either precise deep reactive ion etching (in top‐down approaches)^[^ ^87^ ^]^ or finely controlled atomic layer deposition (in bottom‐up approaches),^[^ ^88^ ^]^ both of which add considerable experimental complexity. In contrast, the nearly planar geometry of our meta‐atoms effectively circumvents these challenges and simplifies the fabrication process.

For the design of the HIRUs for water‐immersed metasurfaces, we employed commercial finite‐difference time‐domain (FDTD) software Lumerical to simulate and analyze the optical properties of candidate meta‐atoms. Periodic boundary conditions were applied along the *x* and *y* axes, as defined in Figure 1c, with linearly polarized light propagating along the positive *z*‐direction (see Experimental Section). All meta‐atoms were simulated on a SiO_2_ substrate (*n_SiO2_* = 1.46) and encapsulated with a 300‐nm‐thick SiO_2_ layer (Section S1, Supporting Information). The surrounding medium was water (*n_water_* = 1.333). During the design process, we combined both forward design, guided by physical insight into multipolar resonances, and heuristic inverse design via PSO. The design objective was to realize visible‐wavelength Huygens’ metasurfaces that satisfy the generalized Kerker condition (GKC), requiring high optical transmission, full phase modulation, and insensitivity to fabrication errors. We began by examining disk‐shaped GaP resonators, simulating diameters ranging from 50 to 300 nm (Figure 2a). These exhibited high transmittance (>75%) and covered a full 2π phase range. We define a term called phase sensitivity to analyze the sensitivity of the meta‐atom's phase response versus the size variation caused by fabrication imperfection. For the reported GaP nanodisk, when the diameter is ~175 nm, ≈a 5‐nm diameter change leads to a ≈50° phase shift, corresponding to a phase sensitivity ≈10°/nm. It indicates that a 1 nm size imperfection will lead to a phase error of 10°. Such an imperfection‐caused phase error will lower the performance of the metasurfaces. In the work, we aim to realize a group of meta‐atoms which show a phase sensitivity less than 9°/nm (i.e., 0.025 π/nm in the corresponding phase sensitivity). Consequently, only three disk geometries with diameters of 194, 210, and 250 nm were selected, labeled as meta‐atom 3, 4, and 5 in Figures 1d and 2a. Next, we evaluated square‐shaped meta‐atoms, which revealed similar fabrication sensitivity. As shown in Figure 2b, rapid phase variation occurred between side lengths of 154–162 nm. Thus, only two square geometries with side lengths of 144 and 152 nm were retained (labeled as meta‐atoms 6 and 7 in Figures 1d and 2b). While the particles located the area that the phase changes relatively faster, the corresponding phase sensitivity for the particles are 3.59 and 7.37, significantly less than the nanodisk counterparts, see Figures S3 and S4 (Supporting Information). To address the remaining phase gap, we simulated cross‐shaped particles, defined as square structures with corner cutouts. By varying arm length and cutout size, we identified two stable configurations: one with arm length 180 nm and cutout size 40 nm (meta‐atom 1, Figure 2c), and another with arm length 200 nm and cutout size 54 nm (meta‐atom 2, Figure 2d). These exhibited phase responses of −150° and −98°, respectively (Figure 1d). To complete the 2π phase range, we turned to integrate PSO into the simulation workflow to perform inverse design (Section S2, Supporting Information) targeting a meta‐atom with high transmittance (>75%), a phase near 167°, all with a fixed height of 85 nm. In the simulation, the minimum linewidth (i.e., the critical dimension) of 50 nm was imposed to ensure the fabrication feasibility of the meta‐atom. While simple geometries such as disks and squares were efficiently optimized through direct parameter sweeps, more complex geometries, where multiple parameters interact nonlinearly, benefit substantially from inverse design methods. PSO is particularly effective in these cases, accelerating the search process in high‐dimensional design spaces and enabling the discovery of configurations that are otherwise difficult to reach through manual exploration. During the optimization, we tested a variety of candidate geometries, including concentric rings, disks surrounded by rings, and other compound shapes. Although some exhibited desirable resonant features, they failed to simultaneously satisfy all key performance metrics. Ultimately, PSO identified a cross‐disk geometry—a cross overlaid on a disk—as a structure capable of meeting all design objectives (labeled as meta‐atom 8 in Figures 1d and 2e). Figure 2e shows the dependence of its optical properties on the arm length of the cross (additional discussion is provided in Section S3, Supporting Information). In summary, we designed a group of eight meta‐atoms, originally starting from a set of GaP nanodisks, with the goal of achieving high transmittance (>75%), full 2π phase coverage with ≈45° phase steps, and strong tolerance to fabrication imperfections. While nanodisks provided good optical performance, most exhibited high phase sensitivity, where minor structural changes (e.g., ±1 nm) could induce significant phase errors. To address this, we systematically replaced five of the nanodisks with alternative geometries, including squares, crosses, and a PSO‐designed cross‐disk, that demonstrated lower phase sensitivity while maintaining the desired optical characteristics. Each of the final eight meta‐atoms exhibits phase sensitivity below our target threshold of 1/40 π per nanometer (≈9°/nm), as detailed in Figure S4 (Supporting Information). In comparison, we analyzed the phase sensitivity of conventional Huygens meta‐atom groups composed solely of commonly used nanodisks (made of TiO_2_ and GaP; see Sections S4 and S5, Supporting Information). The results reveal that our selected HIRUs, which incorporate a variety of geometries, exhibit significantly lower phase sensitivity. This confirms their improved tolerance to fabrication imperfections and supports their suitability for practical implementation in visible‐wavelength water‐immersed metasurfaces.

The large phase modulation and high transmittance observed in the proposed meta‐atoms originate from the strong multipolar resonances supported by their tailored geometries. To validate this, we conducted a comprehensive theoretical analysis of five representative meta‐atom designs, as shown in Figure 3a–e. These geometries closely match those selected from the optimal group highlighted in Figure 2. We first examined the spatial distribution of the electric (*E*) and magnetic (*H*) fields in the meta‐atoms under *x*‐polarized excitation at 532 nm, as presented in Figure 3f–j. Owing to the structural symmetry of the designs, the analysis was restricted to *x*‐polarized excitation; the results are representative, as the symmetry ensures qualitatively similar behavior under *y*‐polarized light, and the metasurface is designed for unidirectional operation. The appearance of field antinodes within the structures indicates strong resonant responses to both electric and magnetic components of the incident light. When multipolar modes in a nanoparticle are spectrally isolated, their scattered fields radiate into both the forward and backward directions, resulting in transmission dips in the spectrum. This occurs because the resonantly scattered light is not phase‐ or direction‐matched to the incident wave, preventing efficient interference and energy transfer in the forward direction. As a result, only a portion of the incident light is coupled through the structure, while the rest is reflected.^[^ ^79^ ^]^ Moreover, such isolated multipolar modes typically lack the ability to induce a full 2*π* phase shift, limiting their usefulness for complete wavefront shaping, as shown in earlier analytical studies.^[^ ^89^ ^]^ In contrast, when the phase and amplitude of multipolar resonances are carefully balanced, near‐field interference among their forward‐scattered fields enables both high transmission and large phase shifts, which are key characteristics for efficient wavefront control. To confirm that the interactions between electromagnetic multipoles in the meta‐atoms lead to highly directional forward scattering, we calculated the generalized Kerker coefficients^[^ ^71^ ^]^ along both forward and backward directions. These coefficients were derived from the induced resonance electric fields corresponding to individual multipolar contributions. We first obtained the strength of each electromagnetic multipole in the meta‐atoms using the multipole decomposition method.

The scattering cross‐sections were obtained by simulating the distribution of electric displacement current and applying the analytical formulations provided in Ref. [90]. The generalized Kerker coefficients for forward ($K_{x}^{FW}$) and backward scattering ($K_{x}^{BW}$) were then calculated using the formulations reported in Ref. [71]:

(1) $$K_{x}^{FW}=\left(p_{x}+ikT_{x}+\frac{m_{y}}{v}+\frac{k}{6i}Q_{xz}+\frac{k}{2vi}M_{yz}\right)$$

(2) $$K_{x}^{BW}=\left(p_{x}+ikT_{x}-\frac{m_{y}}{v}-\frac{k}{6i}Q_{xz}+\frac{k}{2vi}M_{yz}\right)$$

where *p_x_* is the electric dipole, *m_y_* is the magnetic dipole *Q_xz_* is the electric quadrupole, *M_yz_* is the magnetic quadrupole, *v* is the light speed in the medium (*c*/*n_water_*), and *k* is the wavevector (2π(*n_water_*/*λ*)).

The top panels of Figure 3k–o show the amplitudes of the individual multipolar components for the meta‐atoms shown in Figure 3a–e, respectively, while the bottom panels of Figure 3k–o show the corresponding Kerker coefficients. Notably, all meta‐atoms exhibit strong magnetic dipole (MD) responses, with the red curves showing prominent peaks. While the toroidal dipole (TD) also contributes, its amplitude is relatively small compared to the dominant dipolar and quadrupolar components. Despite the strong MD response, which is typically associated with suppressed transmission,^[^ ^91^ ^]^ the total forward‐scattered field (solid curves in the bottom panels of Figure 3k–o) reaches the maximal. This seemingly counterintuitive behavior arises from constructive near‐field interference between the MD and other multipolar components (e.g., ED, EQ, TD), which collectively enhance the forward scattering. We also calculated the phase of the individual multipoles in the meta‐atoms (see Figure S6, Supporting Information), which reveals distinctive phase differences among them. This phase diversity plays a crucial role in enabling destructive interference in the backward direction while promoting constructive interference forward, further boosting transmission. This concept can be supported by the calculation results of the Kerker coefficients. Across all cases, $K_{x}^{FW}$ exhibits significantly higher values than $K_{x}^{BW}$​, confirming that the meta‐atoms support strongly forward‐directed scattering. Furthermore, the $K_{x}^{FW}$ ​for each HIRU peaks near the operational wavelength, indicating optimal mode balancing at the design point. These results confirm that the meta‐atoms satisfy the first‐kind Kerker condition,^[^ ^70^, ^71^ ^]^ providing highly directional forward scattering. For a conceptual discussion of the generalized Kerker condition and how it governs the high transmittance and full phase modulation observed here, see the discussion in Section S8 (Supporting Information). By carefully engineering the geometry of the meta‐atoms, potentially using algorithm‐assisted optimization strategies, the complex interplay of multipolar modes can be finely tuned to achieve both high transmission and full phase control. Representative results for tuned cross and cross‐disk particles used in the metasurfaces can be found in Figure S7 (Supporting Information). In summary, the proposed meta‐atoms exhibit Kerker‐type resonances arising from tailored multipolar interference within each structure. Although the geometrical parameters of the nanoparticles were slightly adjusted in the final metasurface designs to achieve full *2π* phase coverage, the particles retained strong forward scattering and suppressed backward scattering, which is consistent with the Kerker condition. This demonstrates that the high transmittance and large phase modulation achieved across the metasurface stem directly from the underlying multipolar behavior of the meta‐atoms.

## 3 Experimental Demonstration of Water‐Immersed Huygens’ Meta‐Optics

**Figure 4:**
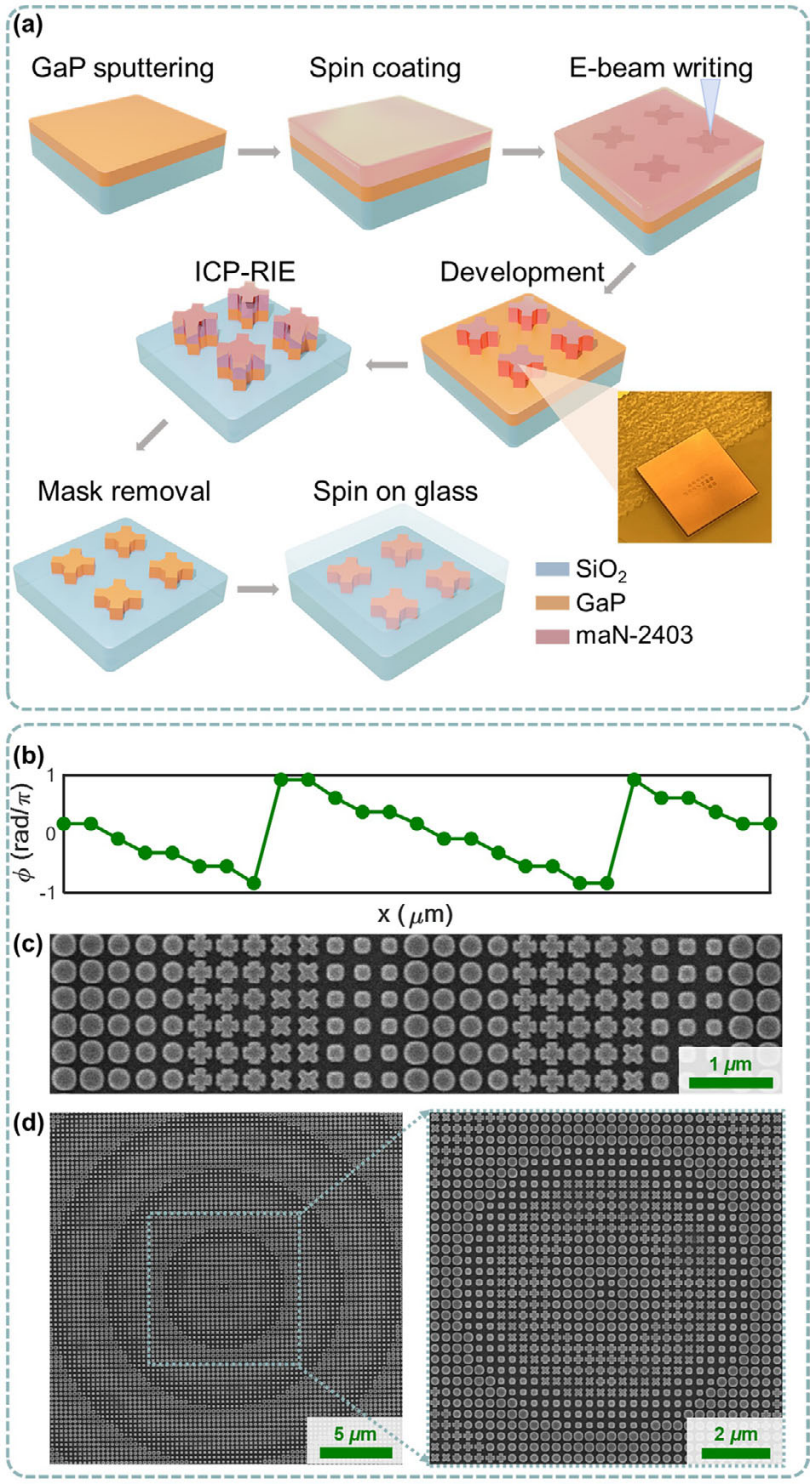
Nanofabrication of the water‐immersed metasurfaces. a) Schematic illustration of the fabrication process flow, including GaP sputtering, resist coating (maN‐2403), electron‐beam lithography, development, ICP‐RIE etching, mask removal, and spin‐on‐glass encapsulation. b) A representative section of the discretized phase profile for a Bessel‐beam‐generating metasurface. c) Zoomed‐in SEM image of the fabricated metasurface. Each Huygens‐integrated resonance unit (HIRU) is spatially arranged to match the phase distribution shown in (b). d) SEM images of the metasurface center under lower magnification, showing the overall phase pattern.

To demonstrate the potential of the proposed HIRUs for realizing functional immersion metasurfaces, we designed and fabricated multiple devices for underwater structured light generation. These metasurfaces were constructed using GaP thin films sputtered on fused silica substrates, patterned via electron‐beam (E‐) lithography and dry etching, following the process flow illustrated in **Figure** **4**a (detailed in Experimental Section). A negative‐tone ma‐N resist was used to define the metasurface patterns, which were subsequently transferred into the GaP layer. The diameter of metasurfaces reported in this paper is 499.84 *µ*m. After fabrication, a 300‐nm‐thick SiO_2_ capping layer was coated atop the metasurfaces using spin‐on‐glass to ensure refractive index symmetry and protect the nanostructures. Due to the planar geometry of the metasurfaces, alternative deposition methods, including plasma‐enhanced chemical vapor deposition and sputtering, can also be employed to make a uniform protection layer coating. As an initial implementation, we developed metasurfaces capable of underwater generation of Bessel and AAF beams. We first fabricated the Bessel‐beam‐generating metasurface. We implemented the phase profile (*ϕ*(*x*,*y*)) to enable the Bessel beam generation of our ultrathin metasurface:^[^ ^57^ ^]^

(3) $$\emptyset \left(x,y\right)=-\frac{2\pi }{\lambda_{eff}}\left(\mathrm{NA}\cdot \sqrt{x^{2}+y^{2}}\right)$$

where numerical aperture (NA) = 0.1, and *λ_eff_* is the effective wavelength in water (532 nm/*n*_water_). The phase profile was subsequently discretized into eight levels with numbers corresponding to the phases of the HIRUs. A cross section of the discretized phase distribution in the resulting phase mask is shown in Figure 4b. The HIRUs were arranged along the substrate surface according to the calculated result and the fabrication process was performed. Scanning electron microscopic (SEM) images of the fabricated metasurface is shown in Figure 4c,d. As can be seen from the magnified SEM image (Figure 4c), each HIRU is spatially arranged and made to match the target phase distribution shown in Figure 4b.

**Figure 5:**
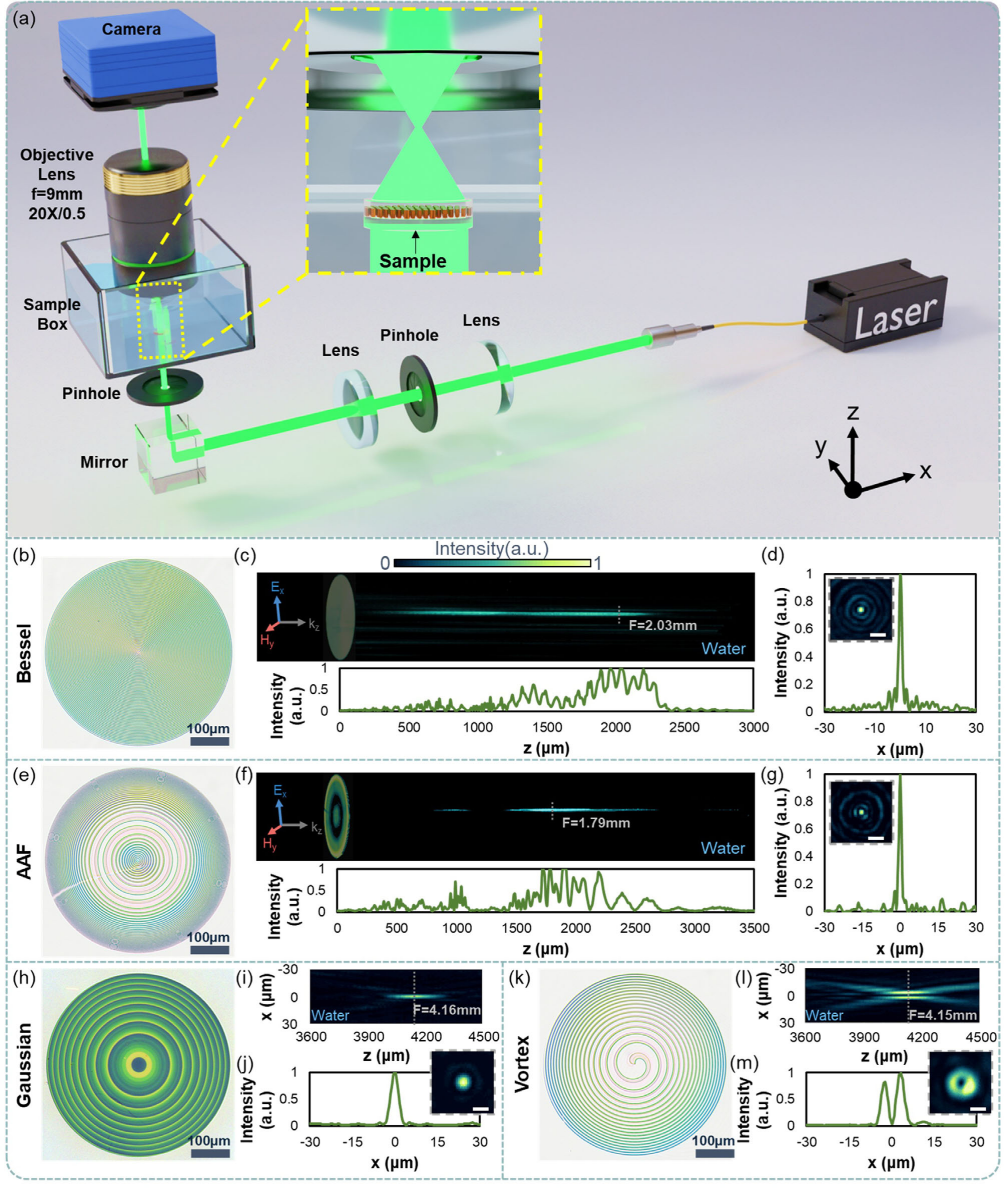
Structured light generation using the water‐immersed HIRU metasurfaces. a) Schematic of the optical setup used for characterizing metasurfaces under full water immersion. b–d) Characterization of the metasurface for generating a Bessel beam: b) Optical image of the metasurface; c) Beam intensity profile along the *z*‐axis (top) and the corresponding cross‐sectional intensity (bottom); d) Cross‐sectional intensity profile at z = 2.03 mm (inset: measured spot image). e–g) Characterization of the metasurface for generating an abruptly autofocusing (AAF) beam: e) Optical image; f) Beam intensity profile along the z‐axis (top) and cross‐sectional intensity (bottom); g) Cross‐sectional intensity profile at *z* = 1.79 mm (inset: focal spot). h–j) Characterization of the metalens for generating a focusing Gaussian beam: h) Optical image; i) Beam intensity profile along the *z*‐axis (top) and cross‐sectional intensity (bottom); j) Cross‐sectional intensity profile at *z* = 4.16 mm (inset: focal spot). k–m) Characterization of the metasurface for generating an optical vortex beam: k) Optical image; l) Beam intensity profile along the *z*‐axis (top) and cross‐sectional intensity (bottom); m) Cross‐sectional intensity profile at *z* = 4.15 mm (inset: focal spot of the FOV beam). Scale bar of the inset in (d,g,j,m): 5 *µ*m.

To characterize the metasurfaces’ properties, a custom‐built system based on an inverted microscope was used. **Figure** **5**a presents a schematic of the system. In the measurement, the metasurface was immersed in water and illuminated by a collimated 532‐nm laser from the substrate side. The output beam was collected using a 20×/0.50 NA water‐immersed objective (Olympus UMPlanFl) and recorded with a camera (Andor Sona). Figure 5b shows the optical image of the Bessel‐beam‐generating metasurface under a broadband white light illumination, where Figure 5c,d show the characterization results by using the 532‐nm laser. The beam propagation (Figure 5c) reveals a non‐diffracting “light sword” beam, a term used in previous metasurface and diffractive optics studies to describe beams with extended axial intensity profiles and long depth of focus,^[^ ^92^, ^93^ ^]^ resembling the propagation behavior of Bessel beams. In our case, the depth of focus (DOF) reaches ≈2.25 mm.

A cross‐sectional view at *z* = 2.03 mm (inset of Figure 5d) shows a central bright spot surrounded by concentric rings, indicative of a first‐order Bessel beam. The measured FWHM is 1.51 *µ*m (standard deviation: 73 nm), closely matching the theoretical value of 1.43 µm. The central peak‐to‐sidelobe intensity ratio exceeds 5:1, showing a large signal contrast between the central spot and the sidelobe.

We further designed a metasurface for generating an AAF beam. Similar to prior designs,^[^ ^22^, ^23^ ^]^ the metasurface introduces a cubic phase term to modulate the incident light into an AAF beam, described by:

(4) $$\emptyset \left(x,y\right)=\frac{{\left(2\pi b_{1}\right)}^{3}{\left(r-r_{0}\right)}^{3}}{3}-\frac{2\pi }{\lambda_{eff}}\left(\sqrt{{\left(r-r_{0}\right)}^{2}+f^{2}}-f\right)$$

where *f* = 1000 *µ*m is the focal length, *r* is the radial distance from the device center ($=\sqrt{x^{2}+y^{2}})$, *b*_1_ = 0.003 *µ*m^−1^ is the attenuation coefficient, *r*_0_ = 100 *µ*m is the main lobe radius of the Airy beam, and *λ_eff_* is the wavelength in water (532 nm/*n_water_*). The fabricated AAF‐generating metasurface is shown in Figure 5e, and its beam propagation characteristics are presented in Figure 5f. A sharp focal spot appears at approximately z = 1500* µ*m. The focal spot features a Bessel‐like intensity profile at the center, as shown in the cross‐sectional view captured at z = 1.79 *µ*m (inset of Figure 5g). This focused beam persists over an extended axial range (up to z ≈ 2500 *µ*m), consistent with previously reported AAF beam behavior. In the early propagation region (z = 0–1000 *µ*m), the beam evolves from a set of concentric hollow rings with low optical intensity, following a parabolic trajectory reminiscent of Airy‐like beams. Although these rings are not distinctly visible in the experimental image due to the high contrast imposed by the intense focal spot, their presence and propagation behavior were confirmed through numerical simulations using the previously reported beam propagation method^[^ ^94^ ^]^ (Figure S8b, Supporting Information). This large intensity contrast between the pre‐focusing region and the sharply localized focal point highlights the defining feature of abruptly autofocusing beams: a sudden and intense light concentration, which is both desirable and consistent with prior demonstrations. Importantly, we also numerically investigated the self‐healing behavior of the Bessel beam generated by the water‐immersed metasurfaces. The Bessel beam was shown to recover a symmetric profile even in the presence of non‐transparent obstructions (Figure S9, Supporting Information). In addition, we confirmed that the hollow structure of the AAF beam enables it to bypass obstacles and still achieve tight focusing downstream (Figure S10, Supporting Information). These properties are especially advantageous for underwater photonic applications, where light must often traverse media containing scattering particles and dynamic obstructions.

To further demonstrate the versatility of the proposed approach for realizing on‐demand water‐immersed metasurfaces, a focusing optical vortex (FOV) generator and, for comparison, a metalens were also fabricated. The phase profile for the water‐immersed metalens was designed by using the parabolic profile.^[^ ^31^ ^]^ The designed numerical aperture and focal length were 0.08 and 4.15 mm, respectively. For the FOV generation, the phase profile was designed by superimposing an azimuthal phase gradient onto the parabolic phase profile, thus enabling for focusing the generated optical vortex at the target focal plane (also 4.15 mm). The surface of the FOV generator was divided into eight angular segments, each with a phase increment of *π*/4 in the clockwise direction in the phase profile.^[^ ^29^ ^]^ Figure 5h,k shows the optical images of the fabricated metalens and FOV generator. The characterization results for the water‐immersed metalens are shown in Figure 5i,j. The measurement shows that the focal length and FWHM of the water‐immersed metalens are 4.16 mm and 3.16 µm at the focus, respectively. The values are close to the theoretical prediction and the theoretical diffraction limit (3.05 *µ*m), respectively. We further analyzed the modulation transfer function (MTF) of the reported water‐immersed metalens and compared the experimental results with theoretical predictions (Figure S12, Supporting Information). The metalens exhibits a cutoff spatial frequency only slightly lower than the theoretical limit, confirming its effective light wave control capability. For the FOV generation, the beam profile and the corresponding cross‐sectional intensity distribution are shown in Figure 5l,m. A donut‐shaped intensity profile can be seen at the focal plane (≈4.15 mm from the device, Figure 5m), a typical characteristic of a focused vortex beam.^[^ ^59^ ^]^ The vortex beam gradually diverges beyond the focal plane, consistent with previous observations of structured beam propagation.^[^ ^59^ ^]^ Furthermore, the mechanical stability of the reported metasurfaces under conditions resembling real‐world immersion environments was evaluated. We performed tests involving vibrational impact in solution. In practical applications such as underwater imaging, biomedical instrumentation, or IoUT systems, devices may be exposed to continuous fluid flow, turbulence, or vibrations induced by mechanical actuation or surrounding equipment. To mimic such conditions in a controlled manner, the device was placed at the bottom of a sonicator's iron container and subjected to continuous sonication in water for 1 h. In this configuration, direct contact between the iron base and the substrate leads to strong mechanical coupling, transmitting vibrational energy directly to the sample. This can result in the detachment or damage of nanostructures on the substrate. For comparison, we also fabricated a TiO_2_ nanopillar array using a previously reported method (Figure S13, Supporting Information),^[^ ^88^ ^]^ with nanopillar diameters comparable to those in earlier visible‐range metalenses.^[^ ^31^ ^]^ For the durability test on the GaP metalens, the sample was subjected to a 1‐h sonication treatment and subsequently immersed in water for seven days. To ensure that the test reflects realistic operating conditions, the vibration energy was delivered through direct substrate coupling rather than indirect bath agitation. As shown in Figure S14 (Supporting Information), the GaP metalens remained structurally intact after sonication, owing to the protection provided by the SiO_2_ layer. In contrast, the reference TiO_2_ nanopillars exhibited significant structural damage immediately after sonication. We note that this test setup was deliberately chosen to challenge the mechanical integrity of the metasurface under harsh conditions. These results demonstrate the superior mechanical stability of the SiO_2_‐coated GaP device under turbulent liquid environments. In addition, we also evaluated the optical performance of the proposed metalens design in several commonly used immersion solutions, including PBS (*n* = 1.335) and oil (*n* = 1.518), to further assess its applicability under different refractive index conditions (Section S14, Supporting Information). The other results on the metalens design with a higher NA can also be found in Figure S15 (Supporting Information). The possibility for the operation of the water‐immersed metalens under oblique illumination was also analyzed (Figure S16, Supporting Information). These results highlight the versatility and robustness of the proposed water‐immersed metasurfaces, demonstrating their potential for advanced underwater photonic applications.

## 4 Conclusion

We have presented water‐immersed metasurfaces composed of GaP‐based Huygens meta‐atoms for structured light generation in the visible regime. By combining multipolar‐resonance physics with inverse design strategies and leveraging the high refractive index of GaP, we realize ultrathin (≈λ/5) metasurfaces that exhibit high transmission, full 2π phase coverage, strong tolerance to fabrication imperfections, and a deeply subwavelength profile. This combination of optical and structural advantages has not been achieved in previously reported immersion metasurfaces. The planar geometry eliminates the need for deep etching typically required in conventional dielectric metasurfaces, and allows the use of protective coatings to improve mechanical stability under flowing water conditions. Building upon these meta‐atoms, we experimentally demonstrate structured light generation spanning the canonical categories of propagation‐invariant beams (e.g., Bessel and abruptly autofocusing beams), optical vortex beams carrying orbital angular momentum, and focusing Gaussian beams. Future integration of intrinsically multi‐resonant or dispersion‐engineered meta‐atoms may further expand the operational bandwidth of these metasurfaces, enabling broader applicability in wavelength‐diverse environments.^[^ ^95^, ^96^, ^97^ ^]^ These metasurfaces effectively replace bulky optical components and operate robustly under full immersion. This platform offers a compact and CMOS‐compatible solution with potential for scalable production.^[^ ^98^, ^99^ ^]^ For instance, our metasurfaces can be fabricated using standard deep ultraviolet (DUV) lithography techniques combined with anisotropic dry etching processes, as demonstrated in recent works.^[^ ^98^, ^100^, ^101^ ^]^ This compatibility with existing high‐throughput semiconductor manufacturing technologies provides a clear path toward volume production. The platform holds strong promise for advancing biomedical processing systems and underwater optical communication. In addition, the demonstrated ability to generate diverse classes of structured light opens new opportunities in underwater optical manipulation, remote sensing, and biophotonics. In the biophotonics domain in particular, these capabilities can benefit compact lightsheet microscopy in confined biological environments,^[^ ^102^ ^]^ nonlinear excitation in microfluidic systems,^[^ ^103^ ^]^ and localized trapping^[^ ^104^ ^]^ or stimulation under immersion conditions. This work paves the way for the development of next‐generation optical components that combine functionality, miniaturization, and environmental robustness for real‐world underwater and biomedical applications.

## 5 Experimental Section

### Design

FDTD‐based commercial software Lumerical was used to simulate and analyze the optical properties of the meta‐atoms used in this paper. In the simulation, periodic boundary conditions were imposed along the *x*‐ and y‐axis. The incident light was sent from the bottom of the unit cells. The optical constants of GaP were acquired by using a spectroscopic ellipsometer (M‐2000, J. A. Woollam). For the multipole expansion analysis, the MATLAB code provided by Ref. [90] was used. In the calculation, the electric displacement current density of the unit cell was calculated and imported into the formulas described in the reference.

### Sample Fabrication

Gallium phosphide (GaP) thin films were sputtered on 500‐µm‐thick fused silica substrates. According to the analysis based on the ellipsometry measurement, the thickness of the GaP thin film was found to be ≈85 nm. The sample was spin‐coated with a 420‐nm‐thick resist, maN‐2403 (Micro Resist Tech.), at a speed of 6000 rpm on top of the GaP layer and prebaked for 1 min at 90 °C. To mitigate the proximity effect due to electron scattering in the resist layer, a conductive polymer ESPACER layer (Resonac) was then spin‐coated atop the sample. E‐beam writing was performed using a Raith Voyager electron‐beam lithography (EBL) system which acceleration voltage, beam current, aperture size, and dose of 50 kV, 60 *µ*m, 300 pA, and 150 µA cm^−2^. The development process was done by immersing the sample in AZ mif700 (Micro Resist Tech.) for 90 s, followed by rinsing the sample in DI water and then carefully dried it with N_2_ blow. Inductively coupled plasma reactive ion etching (ICP‐RIE) was used to transfer the metasurface patterns into the GaP layer, utilizing Cl_2_/H_2_/CH_4_ chemistry with gas flows of 8, 3, and 5.5 sccm, respectively. The ICP and radio frequency (rf) powers were set at 1200 and 100 W, with an operating pressure of 3 mT. Lastly, the residual resist layer was removed by O_2_ plasma. The capping SiO_2_ layers were prepared by using spin‐on glass (NDG‐3000, Desert Silicon). To improve the fidelity of the resulting patterns, proximity effect corrections were applied to the meta‐atom geometries in the GDSII layout files (see Section S21, Supporting Information).

## Conflict of Interest

The authors declare no conflict of interest.

## Author Contributions

J.‐H.L., H.‐Y.W., P.Y.H., and Y.C.C. contributed equally to this work. M.L.T. conceived the idea and designed the research. J.‐ H. Lee and Y. C. Chung performed the PSO in the simulation. H.‐Y.W. and Y.C.C. fabricated the metasurfaces. J.‐H.L. and P.Y.H. designed and analyzed the samples. R.‐H.H. helped with the preparation of the GaP thin films. Y.‐W.H. provided the TiO_2_ sample and assisted with the device design, analysis, and provided advice for the measurement setup. M.L.T. supervised the project. M.L.T., J.‐H.L., P.Y.H., and Y.C.C. wrote the paper.

## Supporting information



Supporting Information

## Data Availability

The data that support the findings of this study are available from the corresponding author upon reasonable request.

## References

[1] J. Huisken , J. Swoger , F. Del Bene , J. Wittbrodt , E. H. K. Stelzer , Science 2004, 305, 1007. 15310904 10.1126/science.1100035

[2] C. J. Bustamante , Y. R. Chemla , S. Liu , M. D. Wang , Nat. Rev. Methods Primers. 2021, 1, 25 34849486 10.1038/s43586-021-00021-6 PMC8629167

[3] B.‐C. Chen , W. R. Legant , K. Wang , L. Shao , D. E. Milkie , M. W. Davidson , C. Janetopoulos , X. S. Wu , J. A. Hammer , Z. Liu , B. P. English , Y. Mimori‐Kiyosue , D. P. Romero , A. T. Ritter , J. Lippincott‐Schwartz , L. Fritz‐Laylin , R. D. Mullins , D. M. Mitchell , J. N. Bembenek , A.‐C. Reymann , R. Böhme , S. W. Grill , J. T. Wang , G. Seydoux , U. S. Tulu , D. P. Kiehart , E. Betzig , Science 2014, 346, 1257998. 25342811 10.1126/science.1257998 PMC4336192

[4] A. Barulin , J. B. Claude , S. Patra , N. Bonod , J. Wenger , Nano Lett. 2019, 19, 7434. 31526002 10.1021/acs.nanolett.9b03137

[5] Z.‐Q. Yan , C.‐Q. Hu , Z.‐M. Li , Z.‐Y. Li , H. Zheng , X.‐M. Jin , Photonics Res. 2021, 9, 2360.

[6] H. Lu , Y. Li , Y. Zhang , M. Chen , S. Serikawa , H. Kim , Mob. Netw. Appl. 22, 2017, 1204.

[7] N. Chi , Y. Zhou , Y. Wei , F. Hu , IEEE Veh. Technol. Mag. 2020, 15, 93.

[8] G. Schirripa Spagnolo , L. Cozzella , F. Leccese , Sensors 2020, 20, 2261. 32316218 10.3390/s20082261 PMC7219055

[9] A. Forbes , M. de Oliveira , M. R. Dennis , Nat. Photonics 2021, 15, 253.

[10] D. McGloin , K. Dholakia , Contemp. Phys. 2005, 46, 15.

[11] D. S. Simon , A Guided Tour of Light Beams: From Lasers to Optical Knots, Iop Concise Physic, UK 2016.

[12] Y. Shen , S. Pidishety , I. Nape , A. Dudley , J. Opt. 2022, 24, 103001.

[13] L. Gao , L. Shao , B. C. Chen , E. Betzig , Nat. Protoc. 2014, 9, 1083. 24722406 10.1038/nprot.2014.087

[14] F. O. Fahrbach , P. Simon , A. Rohrbach , Nat. Photonics 2010, 4, 780.

[15] B. J. Chang , M. Kittisopikul , K. M. Dean , P. Roudot , E. S. Welf , R. Fiolka , Nat. Methods 2019, 16, 235. 30804550 10.1038/s41592-019-0327-9 PMC6561754

[16] A. Paul , A. Volk , M. Hokmabadi , E. Rigo , H. Kermani , L. Almonte‐Garcia , T. A. Finamore , K. M. Iwamoto , R. K. Roeder , G. Timp , Adv. Mater. 2024, 36, 2401344. 10.1002/adma.202401344 38838094

[17] A. E. Carruthers , J. S. Walker , A. Casey , A. J. Orr‐Ewing , J. P. Reid , Phys. Chem. Chem. Phys. 2012, 14, 6741. 22476508 10.1039/c2cp40371d

[18] N. K. Efremidis , Z. Chen , M. Segev , D. N. Christodoulides , Optica 2019, 6, 686.

[19] N. K. Efremidis , D. N. Christodoulides , Opt. Lett. 2010, 35, 4045. 21124607 10.1364/OL.35.004045

[20] M. Manousidaki , D. G. Papazoglou , M. Farsari , S. Tzortzakis , Optica 2016, 3, 525. 10.1364/OL.43.001063 29489781

[21] I. Chremmos , N. K. Efremidis , D. N. Christodoulides , Opt. Lett. 2011, 36, 1890. 21593925 10.1364/OL.36.001890

[22] Y. Luo , M. L. Tseng , S. Vyas , H. Y. Kuo , C. H. Chu , M. K. Chen , H.‐C. Lee , W.‐P. Chen , V.‐C. Su , X. Shi , H. Misawa , D. P. Tsai , P.‐C. Yang , Small Methods 2022, 6, 2101228. 10.1002/smtd.202101228 35212186

[23] J. Hu , Z. Guo , J. Shi , X. Jiang , Q. Chen , H. Chen , Z. He , Q. Song , S. Xiao , S. Yu , N. Chi , C. Shen , Nat. Commun. 2024, 15, 2944. 38580656 10.1038/s41467-024-47105-x PMC10997589

[24] J. Zhang , F. Fan , J. Zeng , J. Wang , Opt. Express 2021, 29, 35570. 34808987 10.1364/OE.442728

[25] A. E. Willner , K. Pang , H. Song , K. Zou , H. Zhou , Appl. Phys. Rev. 2021, 8, 041312.

[26] H. He , M. E. J. Friese , N. R. Heckenberg , H. Rubinsztein‐Dunlop , Phys. Rev. Lett. 1995, 75, 826. 10060128 10.1103/PhysRevLett.75.826

[27] G. Situ , G. Pedrini , W. Osten , J. Opt. Soc. Am. A 2009, 26, 1788. 10.1364/josaa.26.001788 19649113

[28] H.‐H. Hsiao , C. H. Chu , D. P. Tsai , Small Methods 2017, 1, 1600064.

[29] N. Yu , P. Genevet , M. A. Kats , F. Aieta , J.‐P. Tetienne , F. Capasso , Z. Gaburro , Science 2011, 334, 333. 21885733 10.1126/science.1210713

[30] J. Yang , S. Gurung , S. Bej , P. Ni , H. W. Howard Lee , Rep. Prog. Phys. 2022, 85, 036101. 10.1088/1361-6633/ac2aaf 35244609

[31] M. Khorasaninejad , A. Y. Zhu , C. Roques‐Carmes , W. T. Chen , J. Oh , I. Mishra , R. C. Devlin , F. Capasso , Nano Lett. 2016, 16, 7229. 27791380 10.1021/acs.nanolett.6b03626

[32] B. Xu , W. Wei , P. Tang , J. Shao , X. Zhao , B. Chen , S. Dong , C. Wu , Adv. Sci. 2024, 11, 2309648. 10.1002/advs.202309648 PMC11109648 38483885

[33] H. Chung , I. Hwang , J. Yu , G. Boehm , M. A. Belkin , J. Lee , Adv. Sci. 2023, 10, 2207520. 10.1002/advs.202207520 PMC10238174 37029461

[34] R. Sokhoyan , C. U. Hail , M. Foley , M. Y. Grajower , H. A. Atwater , Laser Photonics Rev. 2024, 18, 2300980.

[35] Y. Zhou , T. Zhang , G. Wang , Z. Guo , X. Zang , Y. Zhu , F. Ding , S. Zhuang , Adv. Sci. 2024, 11, 2406571. 10.1002/advs.202406571 PMC11481181 39119949

[36] Y. Lai , D. D. A. Clarke , P. Grimm , A. Devi , D. Wigger , T. Helbig , T. Hofmann , R. Thomale , J.‐S. Huang , B. Hecht , O. Hess , Nat. Commun. 2024, 15, 6324. 39060227 10.1038/s41467-024-50574-9 PMC11282272

[37] G. D. Bai , T. J. Cui , Adv. Sci. 2020, 7, 2001648. 10.1002/advs.202001648 PMC7578880 33101865

[38] K. Konishi , M. Nomura , N. Kumagai , S. Iwamoto , Y. Arakawa , M. Kuwata‐Gonokami , Phys. Rev. Lett. 2011, 106, 057402. 21405435 10.1103/PhysRevLett.106.057402

[39] Z. Tan , F. Fan , S. Guan , H. Wang , D. Zhao , Y. Ji , S. Chang , Adv. Sci. 2023, 10, 2204916. 10.1002/advs.202204916 PMC9896033 36373726

[40] M. V. Gorkunov , A. A. Antonov , A. V. Mamonova , E. A. Muljarov , Y. Kivshar , Adv. Opt. Mater. 2024, 13, 2402133.

[41] Y. Zhou , L. Li , J. Zhang , J. Cheng , X. Liu , Y. Gao , Z. Geng , L. Li , J. Zhou , M. K. Chen , Adv. Sci. 2025, 12, 2412794. 10.1002/advs.202412794 PMC11884584 39806861

[42] Y. Song , J. Yuan , Q. Chen , X. Liu , Y. Zhou , J. Cheng , S. Xiao , M. K. Chen , Z. Geng , PhotoniX 2025, 6, 6.

[43] F. U. Richter , I. Sinev , S. Zhou , A. Leitis , S.‐H. Oh , M. L. Tseng , Y. Kivshar , H. Altug , Adv. Mater. 2024, 36, 2314279. 10.1002/adma.202314279 38511549

[44] T. H. H. Le , A. Morita , K. Mawatari , T. Kitamori , T. Tanaka , ACS Photonics 2018, 5, 3179.

[45] B.‐R. Lee , M. F. Chiang , P. Y. Ho , K.‐H. Chen , J.‐H. Lee , P. H. Hsu , Y. C. Peng , J.‐Y. Hou , S.‐C. Chen , Q.‐Y. Lee , C.‐H. Chang , B.‐R. Li , T.‐E. Lin , C.‐T. Lin , M.‐H. Shih , D.‐H. Lien , Y.‐C. Lin , R.‐H. Horng , Y. Kivshar , M. L. Tseng , Adv. Funct. Mater. 2025, 35, 2420439.

[46] F. Petronella , F. Zaccagnini , M. L. Sforza , V. De Mei , L. De Sio , Adv. Sci. 2025, 12, 2413679. 10.1002/advs.202413679 PMC11884530 39921422

[47] M. Wu , G. Li , X. Ye , B. Zhou , J. Zhou , J. Cai , Adv. Sci. 2022, 9, 2201682. 10.1002/advs.202201682 PMC9353501 35618447

[48] P. Zheng , L. Wu , P. Raj , J. H. Kim , S. K. Paidi , S. Semancik , I. Barman , Adv. Sci. 2024, 11, 2405910. 10.1002/advs.202405910 PMC11615760 39404188

[49] Y.‐H. Chia , W.‐H. Liao , S. Vyas , C. H. Chu , T. Yamaguchi , X. Liu , T. Tanaka , Y.‐Y. Huang , M. K. Chen , W.‐S. Chen , D. P. Tsai , Y. Luo , Adv. Sci. 2024, 11, 2307837. 10.1002/advs.202307837 PMC11132035 38488694

[50] H. Pahlevaninezhad , M. Khorasaninejad , Y.‐W. Huang , Z. Shi , L. P. Hariri , D. C. Adams , V. Ding , A. Zhu , C.‐W. Qiu , F. Capasso , M. J. Suter , Nat. Photonics 2018, 12, 540. 30713581 10.1038/s41566-018-0224-2 PMC6350822

[51] K. Koshelev , S. Kruk , E. Melik‐Gaykazyan , J.‐H. Choi , A. Bogdanov , H.‐G. Park , Y. Kivshar , Science 2020, 367, 288. 31949078 10.1126/science.aaz3985

[52] P. Jangid , F. U. Richter , M. L. Tseng , I. Sinev , S. Kruk , H. Altug , Y. Kivshar , Adv. Mater. 2024, 36, 2307494. 10.1002/adma.202307494 37933748

[53] B. Reineke Matsudo , B. Sain , L. Carletti , X. Zhang , W. Gao , C. de Angelis , L. Huang , T. Zentgraf , Adv. Sci. 2022, 9, 2104508. 10.1002/advs.202104508 PMC9036049 35187854

[54] B. Liu , J. Cheng , M. Zhao , J. Yao , X. Liu , S. Chen , L. Shi , D. P. Tsai , Z. Geng , M. K. Chen , Light: Sci. Appl. 2024, 13, 182. 39107267 10.1038/s41377-024-01530-1 PMC11303724

[55] L. Zhang , S. Mei , K. Huang , C.‐W. Qiu , Adv. Opt. Mater. 2016, 4, 818.

[56] P. C. Wu , W.‐Y. Tsai , W. T. Chen , Y.‐W. Huang , T.‐Y. Chen , J.‐W. Chen , C. Y. Liao , C. H. Chu , G. Sun , D. P. Tsai , Nano Lett. 2017, 17, 445. 27935318 10.1021/acs.nanolett.6b04446

[57] W. T. Chen , M. Khorasaninejad , A. Y. Zhu , J. Oh , R. C. Devlin , A. Zaidi , F. Capasso , Light: Sci. Appl. 2017, 6, 16259. 10.1038/lsa.2016.259 PMC6062187 30167252

[58] W. C. Hsu , C. H. Chang , Y. H. Hong , H. C. Kuo , Y. W. Huang , Nano Lett. 2024, 24, 1808. 38198566 10.1021/acs.nanolett.3c05002

[59] Y. Kim , G. Y. Lee , J. Sung , J. Jang , B. Lee , Adv. Funct. Mater. 2021, 32, 2106050.

[60] P. Huo , C. Zhang , W. Zhu , M. Liu , S. Zhang , S. Zhang , L. Chen , H. J. Lezec , A. Agrawal , Y. Lu , T. Xu , Nano Lett. 2020, 20, 2791. 32155076 10.1021/acs.nanolett.0c00471 PMC7547647

[61] Y. Luo , M. L. Tseng , S. Vyas , T.‐Y. Hsieh , J.‐C. Wu , S.‐Y. Chen , H.‐F. Peng , V.‐C. Su , T.‐T. Huang , H. Y. Kuo , C. H. Chu , M. K. Chen , J.‐W. Chen , Y.‐C. Chen , K.‐Y. Huang , C.‐H. Kuan , X. Shi , H. Misawa , D. P. Tsai , Nanophotonics 2022, 11, 1949. 39633948 10.1515/nanoph-2021-0748 PMC11501894

[62] W. T. Chen , A. Y. Zhu , M. Khorasaninejad , Z. Shi , V. Sanjeev , F. Capasso , Nano Lett. 2017, 17, 3188. 28388086 10.1021/acs.nanolett.7b00717

[63] J. Zhang , H. Liang , Y. Long , Y. Zhou , Q. Sun , Q. Wu , X. Fu , E. R. Martins , T. F. Krauss , J. Li , X. H. Wang , Laser Photonics Rev. 2022, 16, 2200268.

[64] X. Liu , M. K. Chen , C. H. Chu , J. Zhang , B. Leng , T. Yamaguchi , T. Tanaka , D. P. Tsai , ACS Photonics 2023, 10, 2382.

[65] H. Liang , Q. Lin , X. Xie , Q. Sun , Y. Wang , L. Zhou , L. Liu , X. Yu , J. Zhou , T. F. Krauss , J. Li , Nano Lett. 2018, 18, 4460. 29940122 10.1021/acs.nanolett.8b01570

[66] A. Arbabi , Y. Horie , A. J. Ball , M. Bagheri , A. Faraon , Nat. Commun. 2015, 6, 7069 25947118 10.1038/ncomms8069

[67] M. Khorasaninejad , F. Capasso , Nano Lett. 2015, 15, 6709. 26372331 10.1021/acs.nanolett.5b02524

[68] T. Chao , Introduction to Semiconductor Manufacturing Technology, Prentice Hall, USA 2000.

[69] S. Wan , C. Dai , Z. Li , L. Deng , Y. Shi , W. Hu , G. Zheng , S. Zhang , Z. Li , Adv. Sci. 2023, 10, 2205581. 10.1002/advs.202205581 PMC9929123 36529952

[70] W. Liu , Y. S. Kivshar , Opt. Express 2018, 26, 13085. 29801341 10.1364/OE.26.013085

[71] A. Hassanfiroozi , Y. C. Cheng , S. H. Huang , Y. T. Lin , P. S. Huang , Y. Shi , P. C. Wu , Laser Photonics Rev. 2022, 16, 2100525.

[72] L. Zhang , J. Ding , H. Zheng , S. An , H. Lin , B. Zheng , Q. Du , G. Yin , J. Michon , Y. Zhang , Z. Fang , M. Y. Shalaginov , L. Deng , T. Gu , H. Zhang , J. Hu , Nat. Commun. 2018, 9, 1481. 29662052 10.1038/s41467-018-03831-7 PMC5902483

[73] Y. F. Yu , A. Y. Zhu , R. Paniagua‐Domínguez , Y. H. Fu , B. Luk'yanchuk , A. I. Kuznetsov , Laser Photonics Rev. 2015, 9, 412.

[74] A. T. M. Yesilyurt , M. Sanz‐Paz , F. Zhu , X. Wu , K. S. Sunil , G. P. Acuna , J.‐S. Huang , ACS Nano 2023, 17, 19189. 37721852 10.1021/acsnano.3c05649

[75] J. Engelberg , C. Zhou , N. Mazurski , J. Bar‐David , A. Kristensen , U. Levy , Nanophotonics 2020, 9, 361. 10.1364/OL.394680 32667306

[76] M. Decker , I. Staude , M. Falkner , J. Dominguez , D. N. Neshev , I. Brener , T. Pertsch , Y. S. Kivshar , Adv. Opt. Mater. 2015, 3, 813.

[77] I. Staude , A. E. Miroshnichenko , M. Decker , N. T. Fofang , S. Liu , E. Gonzales , J. Dominguez , T. S. Luk , D. N. Neshev , I. Brener , Y. Kivshar , ACS Nano 2013, 7, 7824. 23952969 10.1021/nn402736f

[78] Q. Yang , S. Kruk , Y. Xu , Q. Wang , Y. K. Srivastava , K. Koshelev , I. Kravchenko , R. Singh , J. Han , Y. Kivshar , I. Shadrivov , Adv. Funct. Mater. 2019, 30, 1906851.

[79] A. Howes , W. Wang , I. Kravchenko , J. Valentine , Optica 2018, 5, 787.

[80] C. Gigli , Q. Li , P. Chavel , G. Leo , M. L. Brongersma , P. Lalanne , Laser Photonics Rev. 2021, 15, 2000448.

[81] K. E. Chong , I. Staude , A. James , J. Dominguez , S. Liu , S. Campione , G. S. Subramania , T. S. Luk , M. Decker , D. N. Neshev , I. Brener , Y. S. Kivshar , Nano Lett. 2015, 15, 5369. 26192100 10.1021/acs.nanolett.5b01752

[82] J. Yao , F. Lai , Y. Fan , Y. Wang , S.‐H. Huang , B. Leng , Y. Liang , R. Lin , S. Chen , M. K. Chen , P. C. Wu , S. Xiao , D. P. Tsai , Nat. Commun. 2024, 15, 6543. 39095407 10.1038/s41467-024-50965-y PMC11297327

[83] H. Chen , T. Lin , F. Huang , S. Li , X. Tang , R.‐J. Xie , Adv. Opt. Mater. 2022, 10, 2200836.

[84] A. V. Baranikov , E. Khaidarov , E. Lassalle , D. Eschimese , J. Yeo , N. D. Loh , R. Paniagua‐Dominguez , A. I. Kuznetsov , Laser Photonics Rev. 2024, 18, 2300553.

[85] D. Shima , H. Sugimoto , A. Assadillayev , S. Raza , M. Fujii , Adv. Opt. Mater. 2023, 11, 2203107.

[86] L. Hüttenhofer , M. Golibrzuch , O. Bienek , F. J. Wendisch , R. Lin , M. Becherer , I. D. Sharp , S. A. Maier , E. Cortés , Adv. Energy Mater. 2021, 11, 2102877.

[87] P. S. Huang , C. H. Chu , S. H. Huang , H. P. Su , T. Tanaka , P. C. Wu , Nano Lett. 2023, 23, 10432. 37956251 10.1021/acs.nanolett.3c03056

[88] H.‐T. Su , L.‐Y. Wang , C.‐Y. Hsu , Y.‐C. Wu , C.‐Y. Lin , S.‐M. Chang , Y.‐W. Huang , Nano Lett. 2024, 24, 10055. 39047260 10.1021/acs.nanolett.4c01858 PMC11342354

[89] Nanfang Yu , P. Genevet , F. Aieta , M. A. Kats , R. Blanchard , G. Aoust , J.‐P. Tetienne , Z. Gaburro , F. Capasso , IEEE J. Sel. Top. Quantum Electron. 2013, 19, 4700423.

[90] T. Hinamoto , M. Fujii , OSA Continuum 2021, 4, 1640.

[91] J. C. Ginn , I. Brener , D. W. Peters , J. R. Wendt , J. O. Stevens , P. F. Hines , L. I. Basilio , L. K. Warne , J. F. Ihlefeld , P. G. Clem , M. B. Sinclair , Phys. Rev. Lett. 2012, 108, 097402. 22463666 10.1103/PhysRevLett.108.097402

[92] Z. Zhang , D. Wen , C. Zhang , M. Chen , W. Wang , S. Chen , X. Chen , ACS Photonics 2018, 5, 1794.

[93] B. K. Singh , D. S. Mehta , P. Senthilkumaran , Opt. Lett. 2014, 39, 2064. 24686675 10.1364/OL.39.002064

[94] Y. C. Peng , Y. J. Wang , K.‐H. Chen , Y. H. Lin , H. Sakurai , H.‐C. Chang , C.‐C. Chiang , R.‐T. Duh , B.‐R. Lee , C.‐Y. Huang , M.‐H. Shih , R.‐H. Horng , K. Konishi , M. L. Tseng , Nano Lett. 2025, 25, 3141. 39879353 10.1021/acs.nanolett.4c05552 PMC11869270

[95] R. Lin , J. Yao , Z. Wang , J. Zhou , D. P. Tsai , Laser Photonics Rev. 2024, 19, 2401740.

[96] F. J. Díaz‐Fernández , L. M. Máñez‐Espina , A. Díaz‐Rubio , V. Asadchy , NPJ Nanophoton. 2024, 1, 30.

[97] M. Londoño , A. Sayanskiy , J. L. Araque‐Quijano , S. B. Glybovski , J. D. Baena , Phys. Rev. Appl. 2018, 10, 034026.

[98] J.‐S. Park , S. Zhang , A. She , W. T. Chen , P. Lin , K. M. A. Yousef , J.‐X. Cheng , F. Capasso , Nano Lett. 2019, 19, 8673. 31726010 10.1021/acs.nanolett.9b03333

[99] S. W. Moon , Y. Kim , G. Yoon , J. Rho , iScience 2020, 23, 101877. 33344920 10.1016/j.isci.2020.101877 PMC7736923

[100] A. Leitis , M. L. Tseng , A. John‐Herpin , Y. S. Kivshar , H. Altug , Adv. Mater. 2021, 33, 2102232. 34494318 10.1002/adma.202102232 PMC11468586

[101] T. Hu , C.‐K. Tseng , Y. H. Fu , Z. Xu , Y. Dong , S. Wang , K. H. Lai , V. Bliznetsov , S. Zhu , Q. Lin , Y. Gu , Opt. Express 2018, 26, 19548. 30114125 10.1364/OE.26.019548

[102] T. C. Fadero , T. M. Gerbich , K. Rana , A. Suzuki , M. DiSalvo , K. N. Schaefer , J. K. Heppert , T. C. Boothby , B. Goldstein , M. Peifer , N. L. Allbritton , A. S. Gladfelter , A. S. Maddox , P. S. Maddox , J. Cell Biol. 2018, 217, 1869. 29490939 10.1083/jcb.201710087 PMC5940309

[103] G. Theriault , M. Cottet , A. Castonguay , N. McCarthy , Y. De Koninck , Front. Cell. Neurosci. 2014, 8, 139. 24904284 10.3389/fncel.2014.00139 PMC4032997

[104] Y. Yang , Y.‐X. Ren , M. Chen , Y. Arita , C. Rosales‐Guzmán , Adv. Photonics 2021, 3, 034001.

