# Supplementary material for: Water‐Immersed GaP Huygens’ Meta‐Optics for Visible Structured Light Generation
Source: Adv Sci (Weinh). 2025 Aug 11;12(34):e10467. doi: 10.1002/advs.202510467 (PMC12442711; doi:10.1002/advs.202510467)
Supplement: Supplementary file 1 — Supporting Information [file ADVS-12-e10467-s001.docx]

Supporting Information

Visible GaP Huygens Meta-Optics for Underwater Structured Light Generation

Jia-Hua Lee, Hsing-Yi Wang, Pei Ying Ho, Yu Chia Chung, Ruei-Tzu Duh, Cheng-Ching Chiang, Ray-Hua Horng, Yao-Wei Huang, Ming Lun Tseng*

Content of Supporting Information

1. Influence of the protective layer thickness
2. Particle swarm optimization (PSO)
3. Tolerance to the sample imperfection for the reported unit cell designs
4. Improved phase sensitivity in the modified design
5. Tolerance to the size errors for the GaP and TiO_2_ disk designs
6. Multipole decomposition calculation
7. Multipole phase responses of meta-atoms
8. Generalized Kerker condition (GKC): physical insight into directional scattering
9. Scattering analysis and multipole phase responses of designed meta-atoms
10. Light propagation simulation
11. Beam profile calculation of HIRU metasurfaces
12. Self-healing of metasurface-generated Bessel beam in water
13. Obstacle Bypassing Capability of the AAF Beam
14. Operation in different solvents
15. MTF of the metalens
16. Fabrication of TiO_2_ nanopillars for the robustness test
17. Robustness test of metasurfaces under water immersion
18. Simulation of higher NA metalens
19. Simulated light propagation at various wavelengths
20. Angular tolerance under oblique illumination
21. Proximity effect calibration layouts for HIRUs
22. **Influence of the protective layer thickness**

We tested the dependence of the transmittance of the Huygens’ meta-atom on the thickness of the SiO_2_ capping layer. We take the square-shaped meta-atom with a width of 152 nm as an example. As shown in **Figure S1**, oscillation of the transmittance between 0.86 to 0.93 can be observed. The oscillation is likely due to the Fabry–Pérot interference for the light reflected between the water/SiO_2_ interface. For proper protection of the covered metasurface layer, we choose 300 nm for the fabrication.


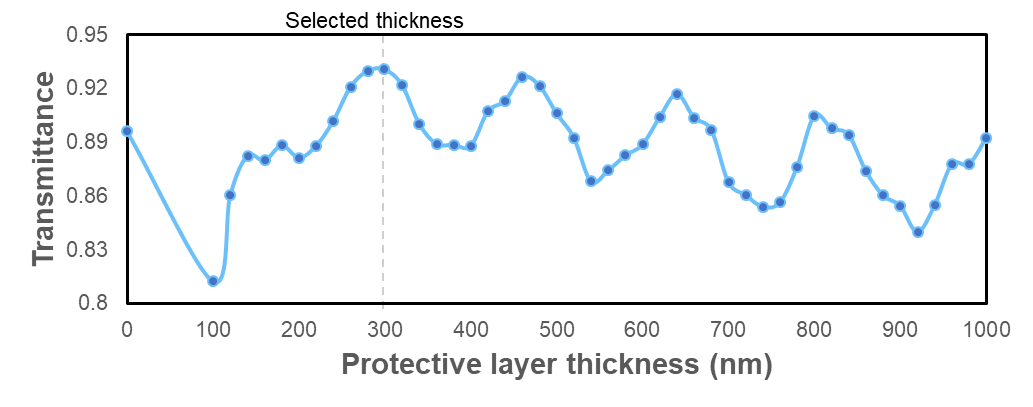


**Figure S1.** **Dependence of the meta-atom’s transmittance on the capping SiO_2_ layer’ thickness. The red dot highlights the maximum transmittance at 300 nm.**

1. **Particle swarm optimization (PSO)**

The Particle Swarm Optimization (PSO) algorithm is a versatile heuristic tool that efficiently explores large solution spaces without requiring extensive problem-specific information, identifying potential solutions through multiple iterations. We applied the PSO algorithm to optimize our unit cell, aiming for a specific phase and desired transmittance as defined by a given objective function. The algorithm was given a range of shape parameters, allowing the unit cell to vary its shape within these limits to find conditions that minimized the objective function. This process ultimately led to the identification of the shield structure. In our design, PSO combined with Lumerical was used to optimize the geometric parameters of the cross-disk in order to achieve the targeted phase modulation necessary for completing the full 2π phase control of the meta-atom group. The calculation process of PSO is shown in **Figure S2**. The optimization objectives included a transmittance greater than 80% and a phase of -74 degrees. The iteration count was set to 20, with the shape designed within a specified range for optimization. The shape design was constrained to ensure axis symmetry, avoid polarization selectivity, and accommodate fabrication limitations, particularly ensuring the feature size was not smaller than the resolution of e-beam lithography. These constraints were managed by carefully setting the range of the resonant unit cell's parameters.


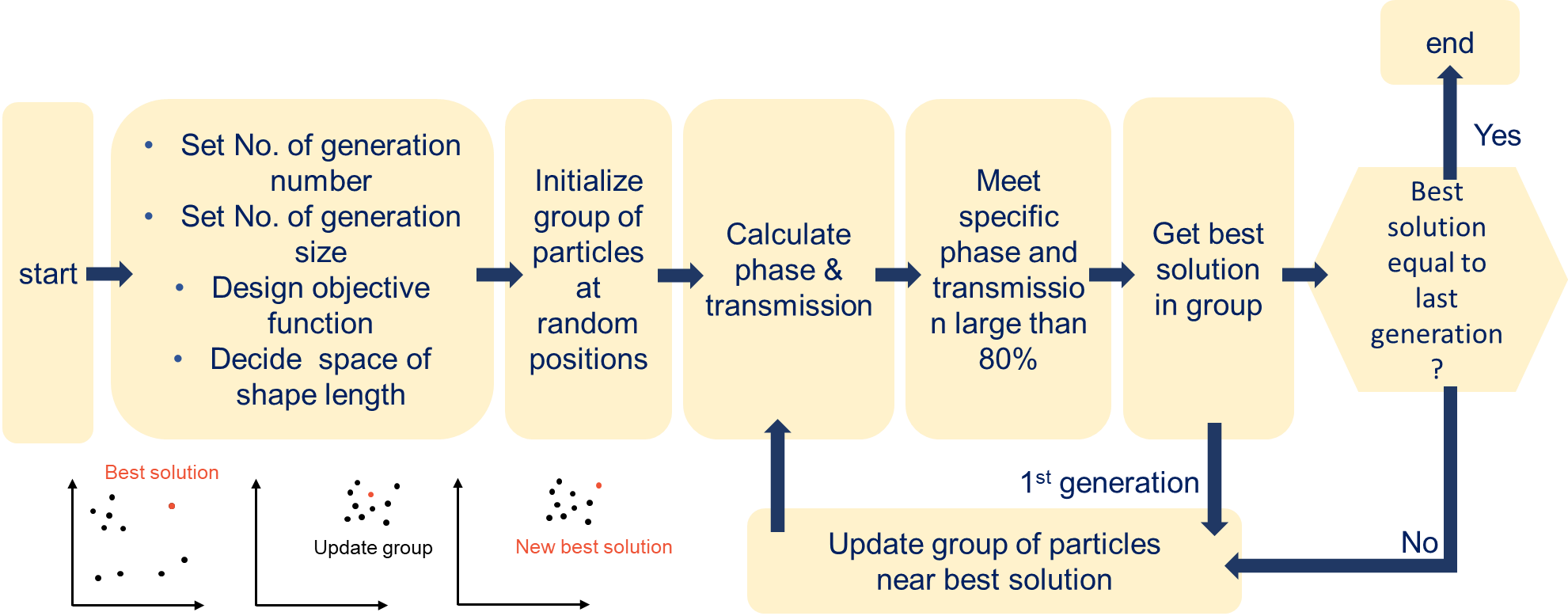


**Figure S2.** **Particle swarm optimization for the meta-atom design.**

1. **Tolerance to the sample imperfection for the reported unit cell designs**

Imperfections in unit cells can significantly degrade the performance of metasurfaces. In this section, we examine the sensitivity of the reported meta-atoms to fabrication imperfections. **Figures S3a-d** illustrate the phase modulation of meta-atoms with various geometric parameters. For better understanding of the change of the phase associated with the size variation, we added a 120-degree phase shift to all the data present in **Figure S3**. For the GaP disks and squares, we analyzed the dependence of phase modulation on their diameter and length, respectively, as size errors are the most common imperfections for these types of meta-atoms. In the case of crosses, achieving precise corners during nanofabrication is crucial. To investigate the relationship between phase and corner cut-out size errors, we defined a parameter, *δ* (shown in the inset of **Figure S3c**), and performed corresponding simulations. For the cross-disk, we evaluated the phase dependence on multiple size parameters, including the length and width of the overlapping cross, as well as the size of the central disk. We applied linear fits to the resulting curves within the parameter ranges used in the final meta-atom design (shown in **Figure 2** in the main text), marking them with blue lines. Overall, the slopes of these fitting lines are less than 9 deg./nm. These results indicate that combining meta-atoms of different shapes can lead to designs that are more tolerant of fabrication imperfections. To further enhance tolerance, additional shapes could be incorporated into the designs.


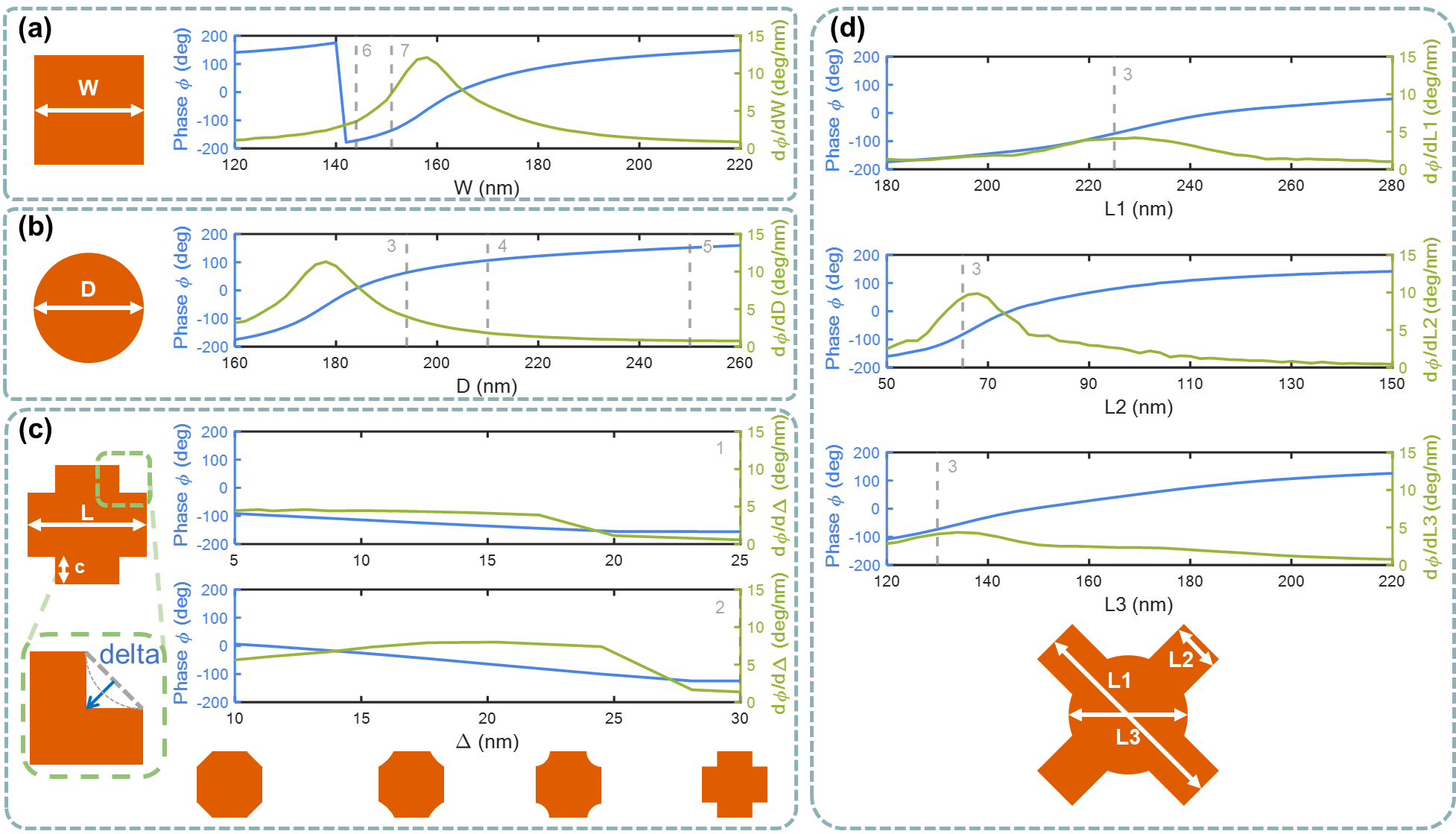


**Figure S3.** **Meta-atoms’ tolerance to the imperfection.** The relationship for the phase versus (a) the length of the square, (b) the diameter of the disk, (c) the cut-out size of the crosses, and (d) the cross’s width and length and the diameter of the overlapping disk in the cross-disk.

1. **Improved phase sensitivity in the modified design**

**Figure S5** shows that the phase sensitivity of the reported unit cell designs composed of different shapes is generally lower compared to the unmodified disks (shown in **Figure S5a**). In **Figure S5a**, the phase sensssitivity reaches a maximum of 11.4 deg/nm when the diameter of the disk is 176 nm. However, in **Figure S4b**, the highest phase sensitivity observed is only 8.91 deg/nm, corresponding to the cross-disk structure. Additionally, most of the non-circular unit cells exhibit lower phase sensitivity values, with some, such as the cross-shaped structure at 180 nm and 200 nm, showing even lower sensitivities of 3.96 deg./nm and 2.56 deg./nm, respectively. The decrease in phase sensitivity may be attributed to the altered light-matter interactions introduced by the more complex geometries.

**
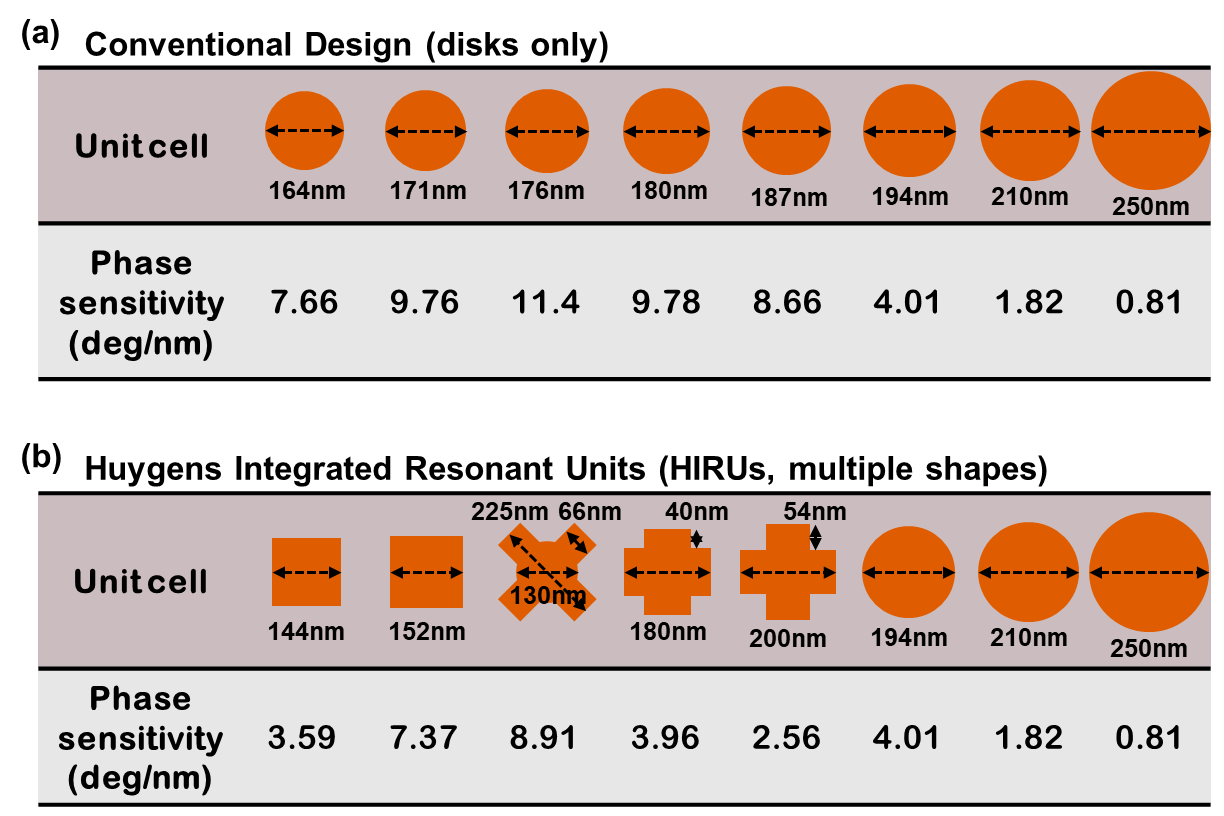
**

**Figure S4.** **Comparison of phase sensitivity between different unit cell designs.** (a) Phase sensitivity of circular unit cells with varying diameters. (b) Phase sensitivity of HIRUs with various geometric shapes, including squares, crosses, and cross-disk structures.

1. **Tolerance to the size errors for the GaP and TiO_2_ disk designs**

**Figure S5a** and **5b** present simulation results showing the relationship between the phase and diameter of TiO_2_ and GaP disks at a wavelength of 532 nm (~364 nm in SiO_2_), respectively. Both TiO_2_ and GaP disks demonstrate a full 2π phase control for transmitted light. However, a rapid phase shift occurs within certain diameter ranges. To analyze this, we introduced a parameter called phase sensitivity, defined as the derivative of the phase with respect to the nanostructure dimension. For TiO_2_ disks, the max phase sensitivity reaches the value of 20.86 deg./nm at the diameter of 186 nm, meaning that a 1-nm fabrication imperfection could result in a 20.86-degree phase error. Achieving unit cell size errors of less than 1 nm in metasurfaces would be highly challenging, making this design impractical for experimental implementation. Similarly, although improved, the GaP disk design also shows a rapid phase change within a specific diameter range (max phase sensitivity ~11.42 deg./nm). As a result, different unit cell shapes were introduced in the metasurface design discussed in the main text.


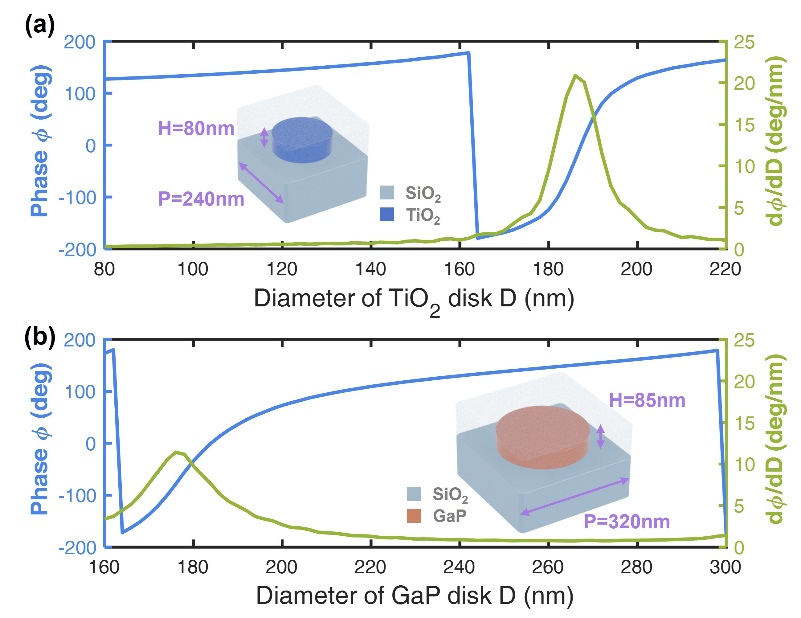


**Figure S5.** **Dependence of the phase on the disk diameter.** (a) The case of TiO_2_ disk. (b) The case of GaP disk.

1. **Multipole decomposition calculation**

We used the open-source MATLAB package MENP, developed by Hinamoto and Fujii,^[1]^ to post-process electric field distributions obtained from Lumerical FDTD simulations. As described in the paper, the induced current distribution J(*r*) was calculated using the relation:

$J\left( r \right)=-i\omega\varepsilon_{0}\left( n^{2}-1 \right)E\left( r \right)$*…...………………………………………………………..…………...(1)*

, where ω, ε_0_, and *n* are the angular frequency, vacuum permittivity, and the refractive index of GaP, respectively. These current distributions were then inserted into MENP to compute the multipole strengths and phases based on exact analytical expressions.

As an example, as described in Ref. [2], the electric dipole moment *p****_α_***​ can be computed using the following formula:

$p_{\alpha}=\frac{-1}{i\omega}\left\{ \int J_{\alpha}j_{0}\left( kr \right)d^{3}r+\frac{k^{2}}{2}\int\frac{\left[ 3\left( \boldsymbol{r}\cdot\boldsymbol{J} \right)r_{\alpha}-r^{2}J_{\alpha} \right]}{\left( kr \right)^{2}}j_{2}\left( kr \right)d^{3}r \right\}$……….…………...….…………...(2)

, where *j_0_* and *j_2_*​ are spherical Bessel functions of order 0 and 2. Similar procedures were used to calculate magnetic dipole and higher-order multipoles. For more detailed theoretical formulations and numerical implementation, please refer to Ref. [1] of Supporting Information.

1. **Multipole phase responses of meta-atoms**

**
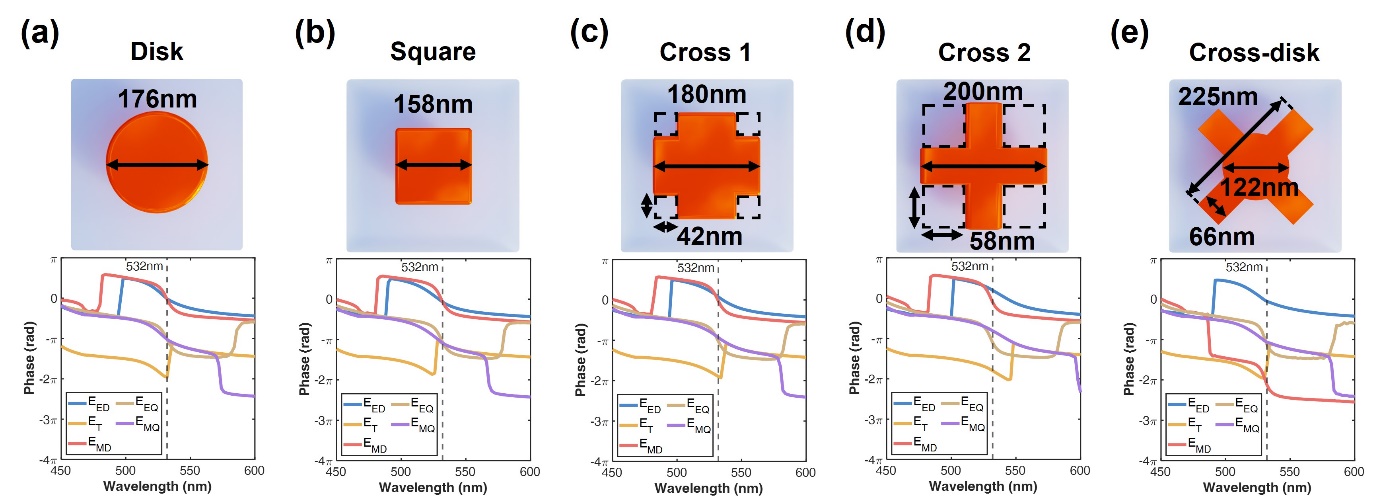
**

**Figure S6.** **Multipole phase responses of meta-atoms with various geometries:** (a) disk, (b) square, (c) cross 1, (d) cross 2, and (e) cross-disk. The top row shows the top-view dimensions of each structure, while the bottom row presents the simulated multipole phase contributions, including electric dipole E_ED_, toroidal dipole E_T_, magnetic dipole E_MD_, electric quadrupole E_EQ_, and magnetic quadrupole E_MQ_.

1. **Generalized Kerker condition (GKC): physical insight into directional scattering**

The first Kerker condition, introduced in 1983 by Kerker *et al.* ^[3]^ describes a situation in which light scattered by a particle is predominantly directed forward, with negligible backscattering. Originally formulated for hypothetical magnetic spherical particles with coexisting electric and magnetic dipole moments, this condition is met when the dipole amplitudes are equal and oscillate in phase. In modern nanophotonics, this principle has been generalized to dielectric or semiconductor nanostructures, where even nonmagnetic materials can exhibit magnetic dipole-like behavior due to displacement current loops. In the generalized Kerker condition (GKC) framework,^[4-5]^ higher-order multipoles such as electric and magnetic quadrupoles and toroidal dipoles are also considered. By engineering constructive interference in the forward direction and destructive interference in the backward direction among these multipoles, metasurfaces can achieve high transmittance and full 2π phase modulation. This mechanism is critical for achieving high-performance, ultrathin, immersion-compatible metasurfaces without relying on Fabry–Pérot propagation modes or high-aspect-ratio geometries. In this work, the proposed GaP meta-atoms are carefully designed to satisfy the GKC, as verified by calculated multipolar scattering amplitudes and directional Kerker coefficients (see **Figures 3** and **S7**). This design enables strong forward scattering, minimized reflection, and robust operation in aqueous environments.

1. **Scattering analysis and multipole phase responses of designed meta-atoms**

**
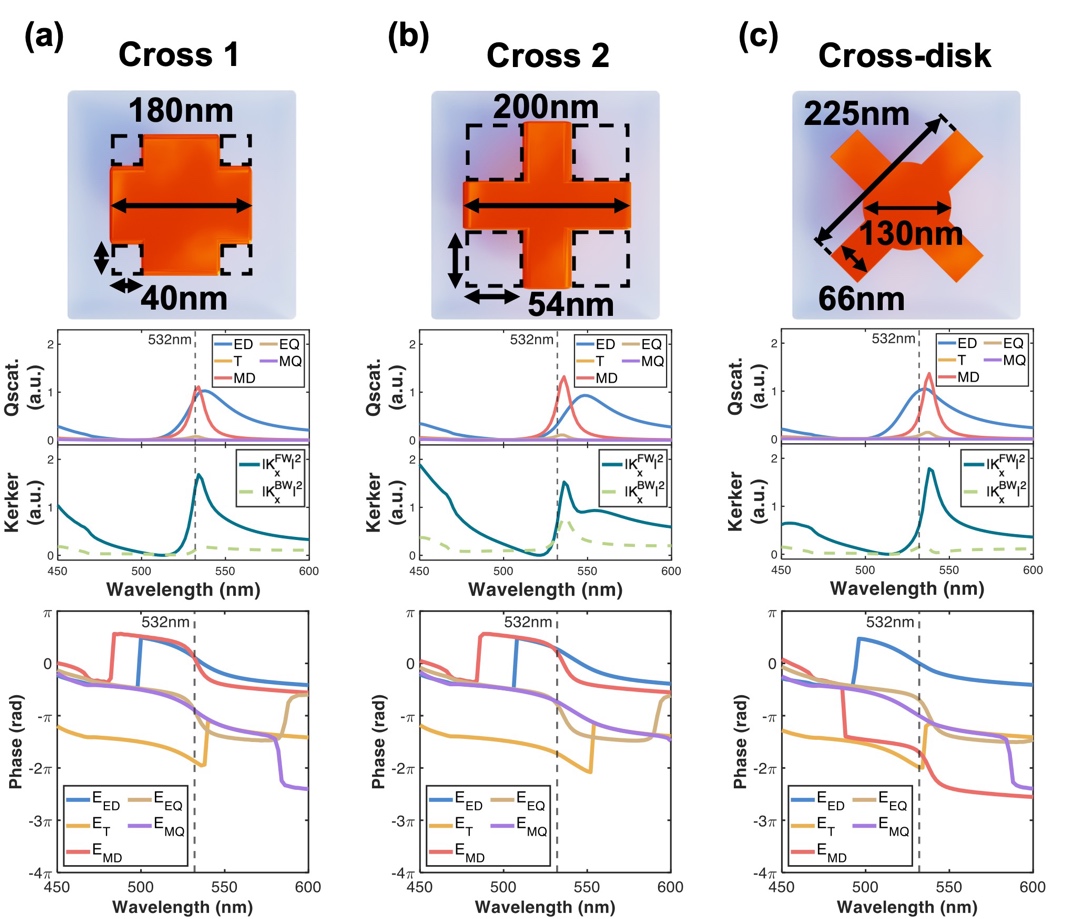
**

**Figure S7.** **Geometries and corresponding optical responses of three designed meta-atoms:** (a) cross 1, (b) cross 2, and (c) cross-disk. The top row shows the top-view dimensions of each structure. The middle row presents the calculated scattering cross section Q_scat_ and Kerker coefficients $\left| K_{x}^{FW} \right|^{2}$ and $\left| K_{x}^{BW} \right|^{2}$. The bottom row displays the multipole phase contributions, including electric dipole E_ED_, toroidal dipole E_T_, magnetic dipole E_MD_, electric quadrupole E_EQ_, and magnetic quadrupole E_MQ_.

1. **Light propagation simulation**

We used a MATLAB modulate based on the beam propagation method to simulate the propagation properties of the metasurfaces reported in this paper. The method has been detailed in the previous paper. In short, it was modeled as a circular field mask, which the diameter corresponds to the metasurface made in the paper. The field mask is meshed into rectangular grids with a length of 320 nm, corresponding to the period of the meta-atoms. Each grid represents a GaP meta-atom with encoded transmission and phase properties, while regions outside the aperture were set to zero amplitude. Plane wave illumination was used, and wavefront propagation was calculated iteratively via FFT under the Fresnel approximation. Longitudinal wavevectors determined phase shifts, with IFFT reconstructing spatial-domain fields. Monitors placed along the optical axis retrieved key properties, including focal length, spot size, and point spread function, demonstrating the metalens' optical performance.

1. **Beam profile calculation of HIRU metasurfaces**

We used the previously reported beam propagation method (BPM) simulation^[6]^ to calculate the propagation properties of the structured light beams generated by our HIRU water-immersion metasurfaces. The metasurfaces were represented as phase masks on a discretized Cartesian grid (The leftmost panels of **Figures S8a-d**), with each grid point corresponding to a HIRU. A plane wave was used as the input source, and the complex field distribution was initialized accordingly. Light propagation was simulated using a scalar diffraction model under the Fresnel approximation, employing fast Fourier transforms (FFT) to compute the wavefront evolution in the frequency domain. Inverse FFT was applied at each step to retrieve the spatial field distribution along the optical axis. This approach enabled the extraction of key characteristics such as focal distance and point spread function from the resulting field profiles at various propagation planes.

**
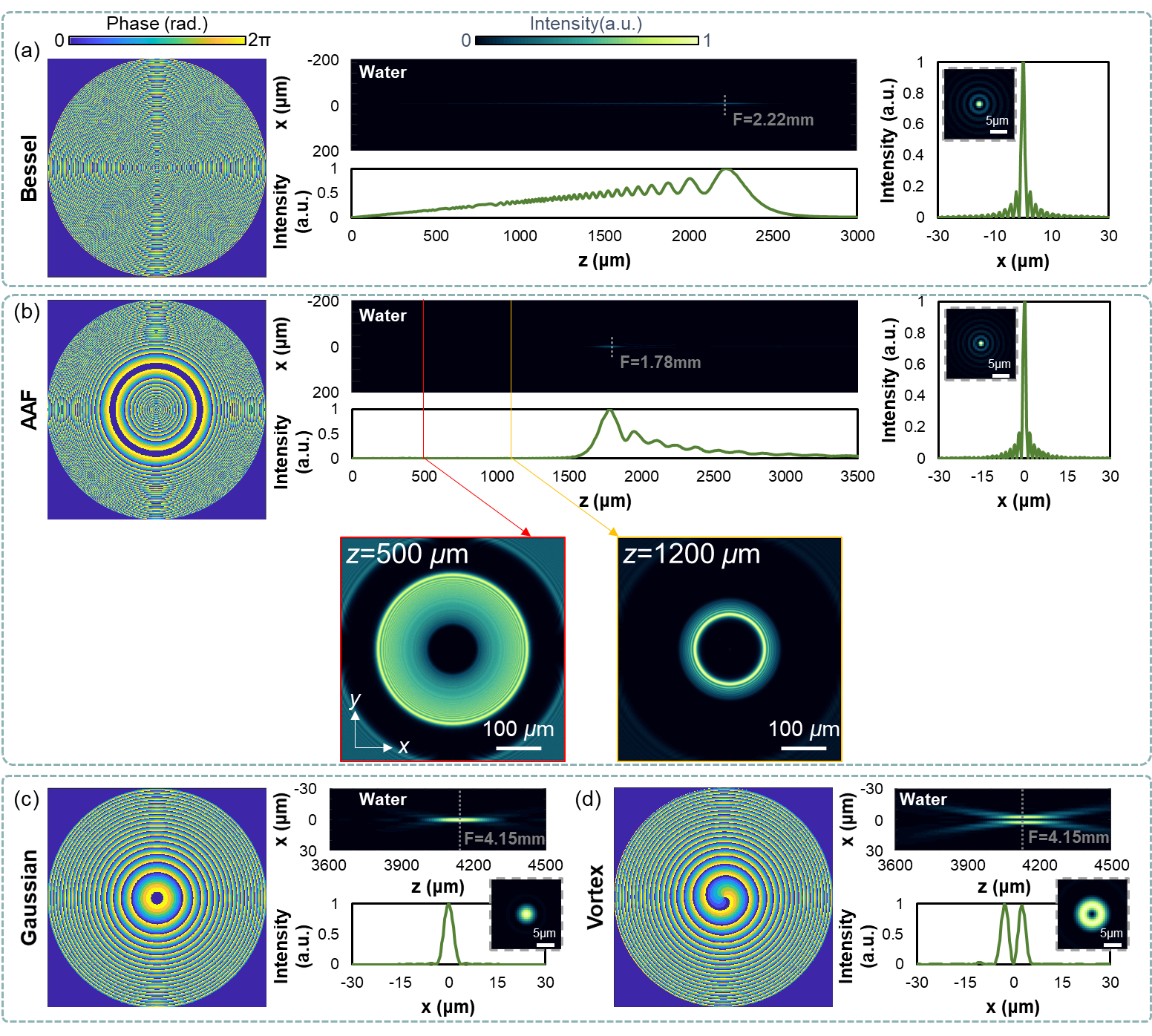
**

**Figure S8.** **Calculated characterization of** **the immersed metasurfaces** **for generating** (a) Bessel beam, (b) AAF beam, (c) Gaussian beam, and (d) vortex beam.

1. **Self-healing of metasurface-generated Bessel beam in water**

Bessel beams possess a unique wave structure that enables them to reconstruct their profile after partial obstruction, maintaining a symmetric beam spot beyond the obstacle^[7]^. To verify that the Bessel beam generated by the reported HIRU metasurface exhibits this self-healing property, we designed a corresponding metasurface and simulated its propagation behavior in water, both with and without an obstacle. Due to computational limitations, the metasurface size was reduced to 10 μm, and the numerical aperture (NA) was adjusted to 0.2 for full-wave simulations using Lumerical. The obstacle was modeled as a non-transparent perfect electric conductor (PEC) disk with a diameter of 120 nm and a thickness of 500 nm. **Figure S9a** (top panel) shows the generated Bessel beam in water, forming a symmetric spot at z = 14 μm (bottom panel). When the obstacle was placed along the propagation path at z = 12 μm, the beam exhibited noticeable disruption (**Figure S9b, top**). Nevertheless, the beam reconstructed itself and regained a symmetric profile at z = 14 μm (**Figure S9b, bottom**). A similar behavior was observed when the obstacle was laterally shifted 200 nm to the left: the beam was initially perturbed but rapidly recovered downstream of the obstruction (**Figure S9c, bottom**). These results confirm that the HIRU metasurface-generated Bessel beam demonstrates robust self-healing capabilities in aqueous environments, making it well-suited for operation in particle-rich or flowing media.

**
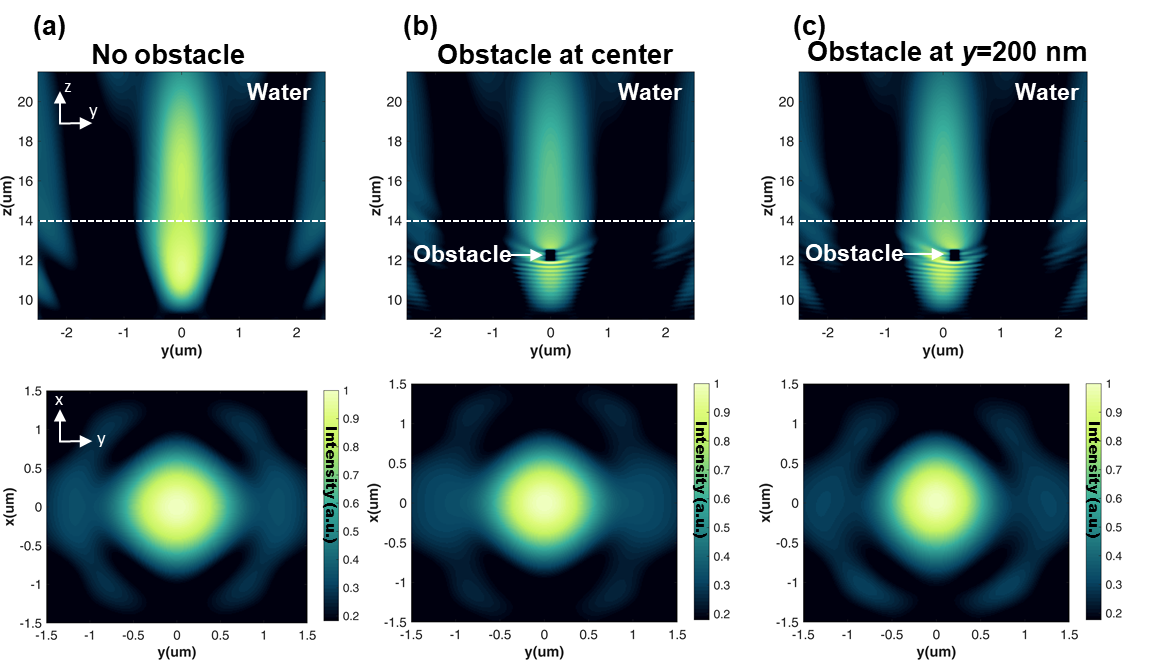
**

**Figure S9. Self-healing of the** **water-immersion HIRU metasurface generated Bessel beam.** (a) Simulation results without an obstacle. Top: beam profile in the *x*–*z* plane. Bottom: beam profile in the *x*–*y* plane at *z* = 14 μm. (b) Simulation results with an obstacle placed at (*x*, *y*, *z*) = (0, 0, 12.25 μm). Top: beam profile in the *x–z* plane showing beam disruption. Bottom: restored beam profile in the *x–y* plane at *z* = 14 μm. (c) Simulation results with the obstacle shifted laterally to (*x*, *y*, *z*) = (0, 0.2 μm, 12.25 μm). Top: beam profile in the *x*–*z* plane. Bottom: recovered beam profile in the *x*–*y* plane at *z* = 14 μm.

1. **Obstacle Bypassing Capability of the AAF Beam**

Abrupt autofocusing (AAF) beams exhibit distinctive hollow beam profiles prior to their sudden focusing at a designated target plane. This unconventional spatial structure enables them to bypass obstacles positioned along their propagation path^[8]^. To validate that the AAF beam generated by the HIRU metasurface exhibits such obstacle-bypassing behavior, we designed a corresponding metasurface and simulated its propagation in water, both with and without an obstacle. Due to computational constraints, the metasurface size was reduced to 35 μm. The design parameters were set to *f*=250 μm, *r_0_*=6 μm, and *b_0_*=0.003 μm^−1^ for full-wave simulations performed in Lumerical. The obstacle was modeled as a non-transparent perfect electric conductor (PEC) disk with a diameter of 10 μm and a thickness of 500 nm. **Figure S10a** shows the simulation setup in the software, and the resulting AAF beam propagation in water is presented in **Figure S10b**. The metasurface successfully generates an AAF beam with a bright focal spot around z≈190 μm. Notably, when the obstacle is positioned close to the metasurface at z=0.75 μm (**Figure S10c**), the resulting AAF beam still maintains a comparable focusing profile at the target plane (**Figure S10d**). These results confirm that the AAF beam generated by the HIRU metasurface is capable of bypassing obstacles in aqueous environments, highlighting its robustness for operation in particle-rich or dynamic fluidic conditions.

**
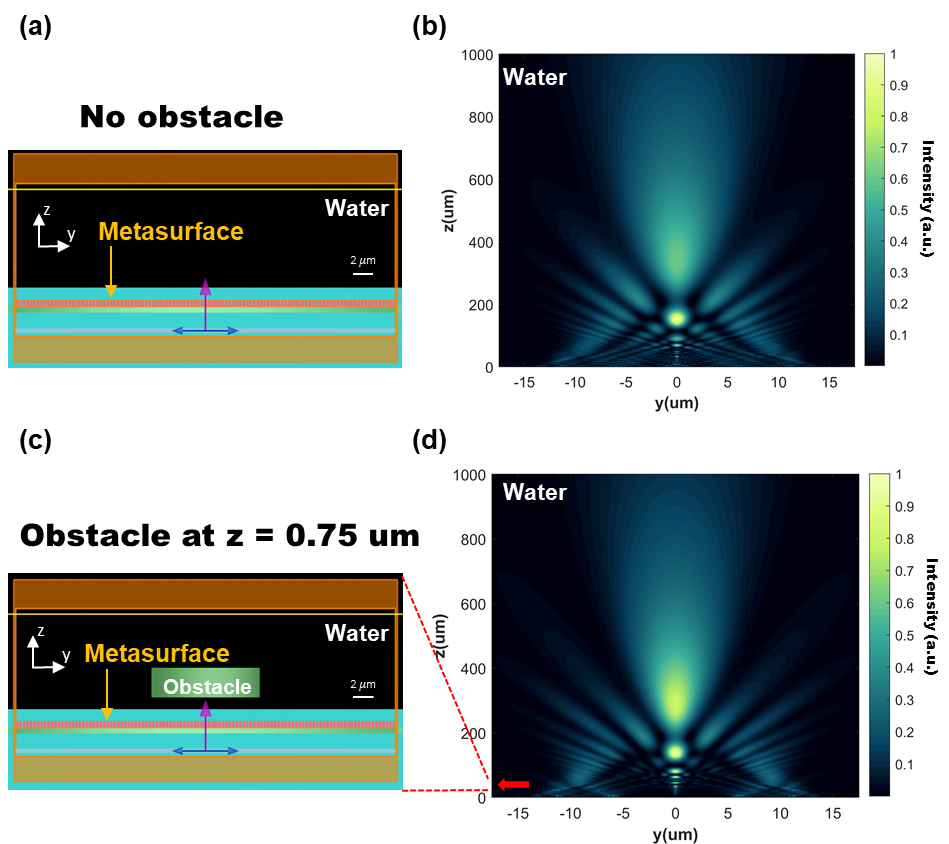
**

**Figure S10. Obstacle bypassing behavior of the AAF beam generated by the HIRU metasurface.** (a) Simulation setup of the AAF-generating HIRU metasurface immersed in water. (b) Simulated beam profile in the x–z plane without the obstacle. (c) Simulation setup with a PEC obstacle placed near the metasurface. (d) Simulated beam profile in the x–z plane in the presence of the obstacle, showing minimal disruption to the focusing behavior.

1. **Operation in different solvents**

**Figure S11** presents simulated beam propagation profiles for a metalens immersed in different media. The metalens has a numerical aperture (NA) of 0.15 and a diameter of 14.4 μm. The focal length (F) varies with the refractive index of the immersion medium. These results were obtained using FDTD (finite-difference time-domain) simulations on a small-area metalens to analyze its focusing behavior. Due to computational limitations, the simulation was restricted to this compact lens size; nevertheless, the results remain representative and offer meaningful insight into the response of change of the surrounding solution. Notably, because each HIRU meta-atom is fully covered by a 300-nm-thick protective SiO_2_ layer, the immediate dielectric environment around the resonators remains nearly unchanged across different immersion conditions. As a result, the multipolar resonances and corresponding phase response at the meta-atom level are preserved. The observed focal length shift thus primarily arises from the change in effective wavelength in the immersion medium, rather than from tuning of the optical resonance in the metasurface.

**
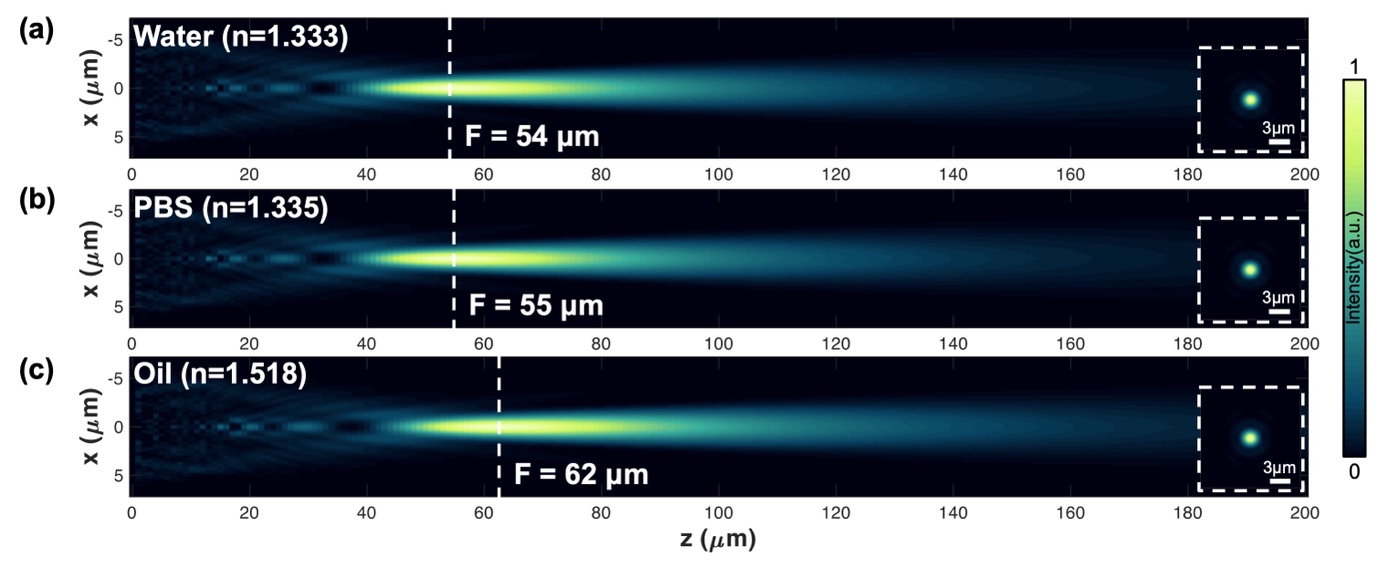
**

**Figure S11.** **Beam propagation profiles in different immersion media.** (a) water (*𝑛*=1.333), (b) phosphate-buffered saline (PBS, *𝑛*=1.335), and (c) oil (*𝑛*=1.518). The insets in the right corners display the focal spot distributions.

1. **MTF of the metalens**

**Figure S12** shows the metalens’ focal spot and the associated analysis of the line spread function and MTF.


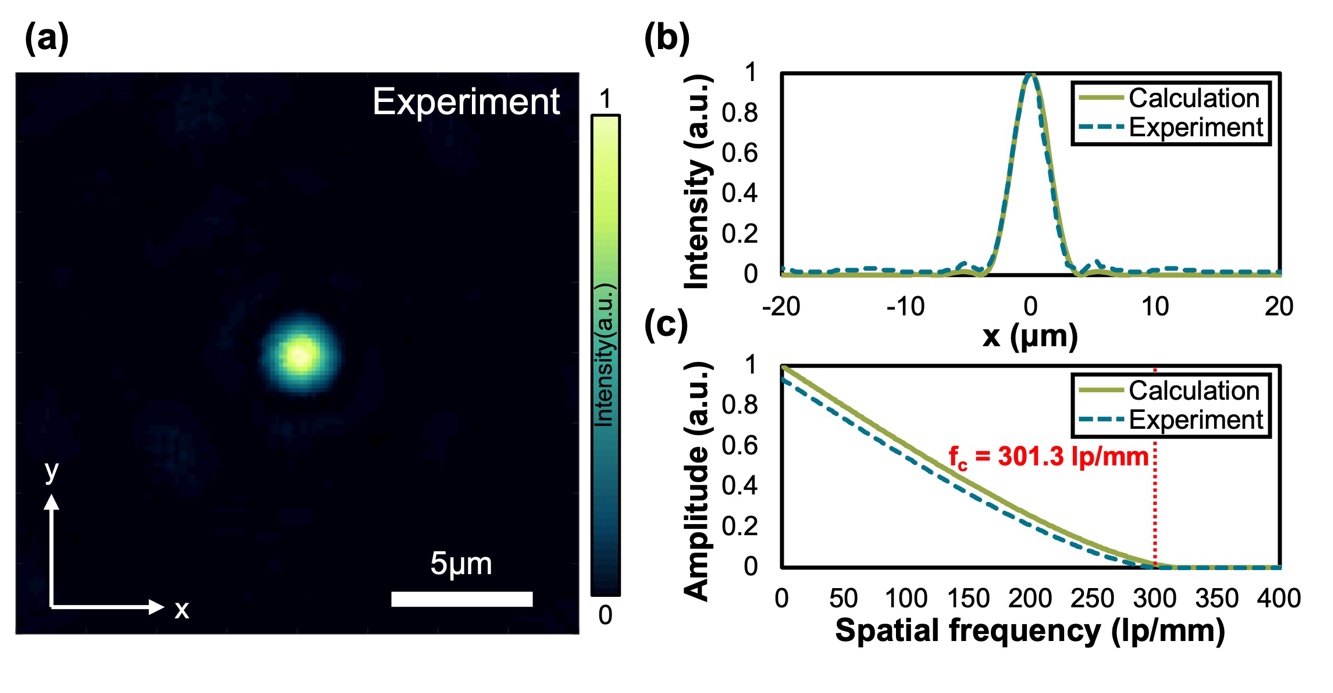


**Figure S12. MTF of the metalens.** (a) Focal spot of the fabricated metalens and the corresponding (b) line spread function and (c) MTF analysis. lp/mm: line pairs per millimeter.

1. **Fabrication of TiO_2_ nanopillars for the robustness test**

To fabricate high aspect-ratio TiO₂ meta-atoms, a bottom-up atomic layer deposition (ALD) technique is employed^[9]^. As shown in **Figure S13**, the process begins by spin-coating a 160-nm-thick electron beam resist (EBR) ZEP onto a fused silica substrate, followed by a conductive layer (Espacer Z300) to enhance electron beam lithography (EBL) uniformity. The EBL system patterns the resist, and the exposed regions are developed to form inverse structures. TiO₂ is then deposited using ALD at 90°C to maintain its amorphous form and prevent EBR damage. Precursors TDMAT and H₂O are alternately introduced until the gaps are filled, requiring a minimum deposition thickness of wₘₐₓ/2, where wₘₐₓ is the widest feature. After deposition, excess TiO₂ is removed by RIE, which is straightforward due to the planar surface. Finally, the EBR and residues are stripped, completing the meta-atom structures for the metasurface waveguide grating (MRWG).

**
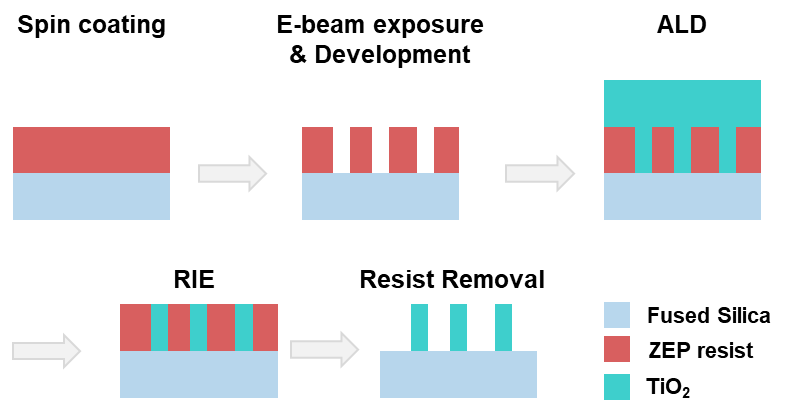
**

**Figure S13.** **Process for fabricating TiO_2_ nanopillars.**

1. **Robustness test of metasurfaces under water immersion and sonication**

This study investigates the mechanical robustness of nanostructured metasurfaces under prolonged exposure to aqueous environments and vibrational stress. To assess structural reliability, all samples were subjected to an ultrasonic cleaning procedure for 1 hour, followed by immersion in deionized water for 7 days. The left column of Figure S14 presents SEM images of TiO₂ nanopillars with a height of 600 nm, fabricated using a standard dry etching process. After sonication, the nanopillars exhibited significant structural deformation and collapse, indicative of their mechanical fragility. In contrast, the right column shows GaP metasurfaces coated with a 300-nm-thick SiO₂ protection layer. In this robustness test, the SiO₂ layer was deposited using plasma-enhanced chemical vapor deposition, a method that provides conformal dielectric encapsulation and strong mechanical bonding to the nanostructured surface. After the same sonication and immersion treatment, the GaP metasurfaces retained their structural integrity with no observable damage. These results confirms the importance of nanostructure geometry and encapsulation in enhancing environmental durability. Compared to conventional high-aspect-ratio TiO₂ nanopillars, our GaP-based metasurfaces with lower aspect ratio designs and dielectric encapsulation demonstrate significantly improved mechanical resilience under realistic immersion conditions.

**
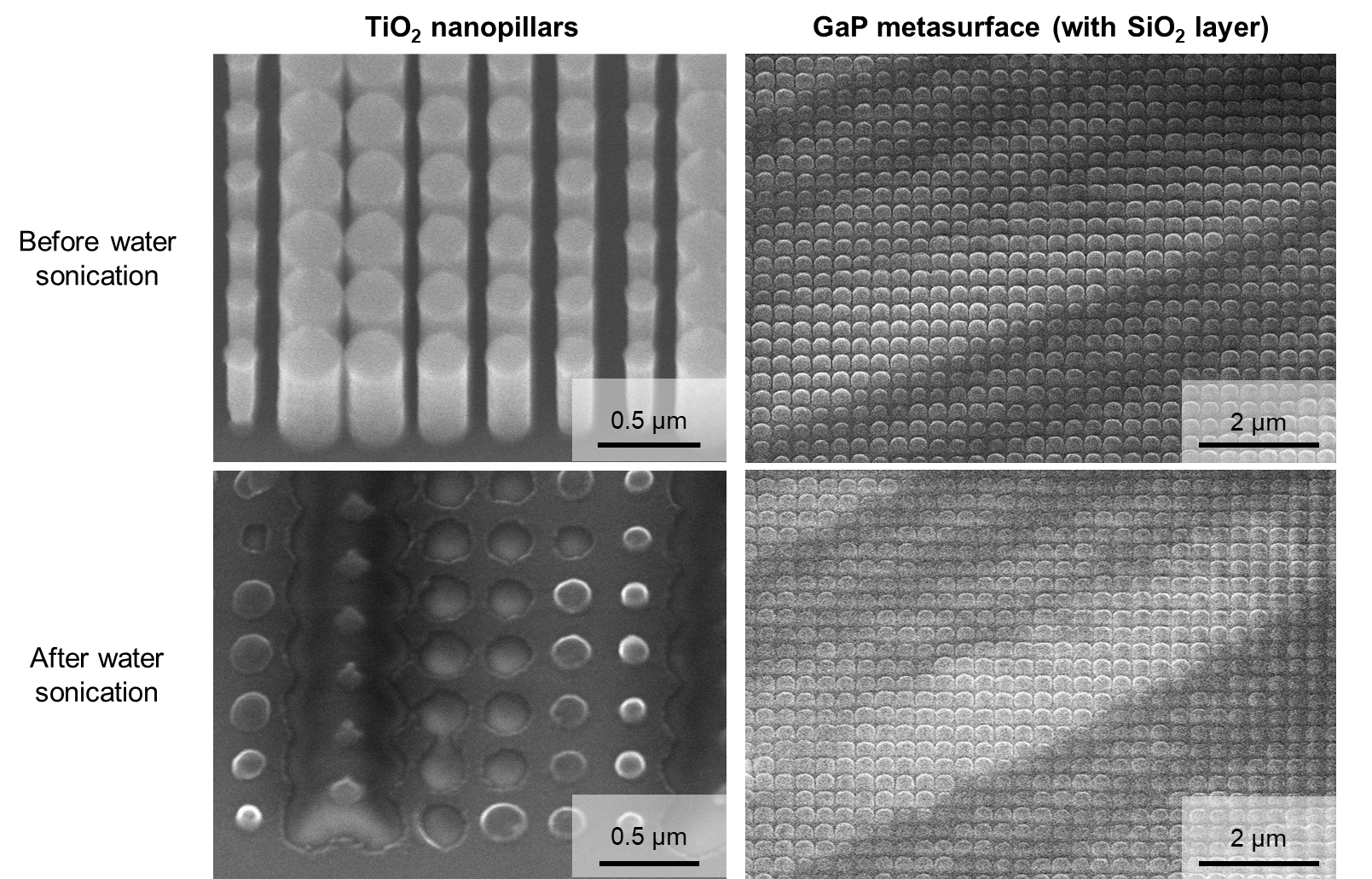
**

**Figure S14. Robustness test of metasurfaces.** SEM images comparing the robustness of TiO_2_ nano-pillars (left) and our low aspect ratio GaP nanostructures (right) before (top) and after (bottom) water immersion.

1. **Simulation of higher NA metalens**

**Figure S15** presents the focal spot and intensity distribution along the optical axis for a high numerical aperture (NA) metalens, designed with NA = 0.8. The metalens, simulated using the FDTD method, has a small diameter of 14.4 μm. The focal length is determined to be 9.4 μm, as indicated by the dashed white line. The inset shows the corresponding focal spot profile.

**
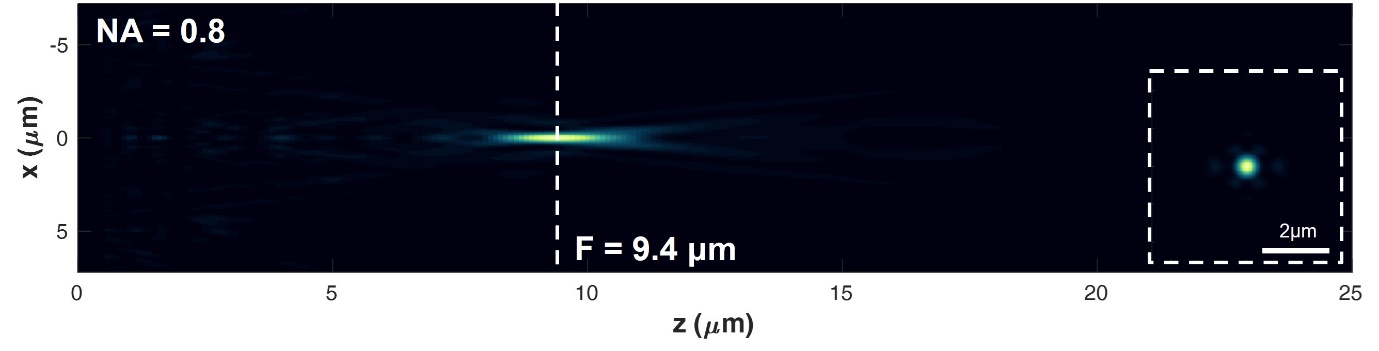
**

**Figure S15.** **Beam propagation profiles** **of a metalens with NA = 0.8.**

1. **Angular tolerance under oblique illumination**

To evaluate the angular tolerance of the proposed water-immersed metalens, we performed numerical simulations under oblique plane wave illumination. The incident angle was varied from 0° (normal) to 5° and 10°, while maintaining linear polarization along the *x*-direction. The simulation was conducted at a wavelength of 532 nm, using a metalens composed of the reported HIRUs with a diameter of 30 µm and a numerical aperture (NA) of 0.07. In the simulation, the incident light was propagating along the positive *z* direction. Due to computational limitations, the simulation was restricted to this compact lens size; nevertheless, the results remain representative and offer meaningful insight into the angular response of the design. **Figure S16** presents the simulated intensity profiles along the *x*–*z* propagation plane.

As shown in **Figure S16a**, the metalens produces a clear and concentrated focal spot under normal incidence, consistent with design expectations. At 5° incidence (**Figure S16b**), the lens still generates a converging beam, though the focal intensity is reduced and additional sidelobes appear. At 10° (**Figure S16c**), the focusing behavior is significantly degraded and becomes spatially diffuse. These results confirm that the proposed metasurface operates optimally under near-normal illumination and becomes less effective at large incidence angles. This limitation arises from the angular sensitivity of resonance-based HIRU meta-atoms, where off-axis excitation modifies the distribution of excited multipoles and introduces phase mismatches. This leads to deterioration of the wavefront quality and degradation of the beam focus.

We note that in most envisioned applications such as fluorescence excitation, underwater imaging, or optical trapping, the system setup typically ensures well-aligned and collimated beams. Minor angular misalignments can be corrected using customized mechanical alignment structures. As a future direction, recent studies have suggested that metasurface designs dominated by specific higher-order multipolar modes, such as electric or magnetic quadrupoles, may offer improved angular robustness.^[10]^ While the current HIRU designs leverage a combination of multipolar contributions (including electric dipole, magnetic dipole, electric quadrupole) to achieve the generalized Kerker condition, future iterations could explore structures in which high-order quadrupolar resonances are deliberately enhanced to suppress angular dispersion. Incorporating such characteristics into the meta-atom designs, potentially through inverse design strategies, may offer a pathway toward metasurfaces with expanded angular tolerance under immersion conditions.

**
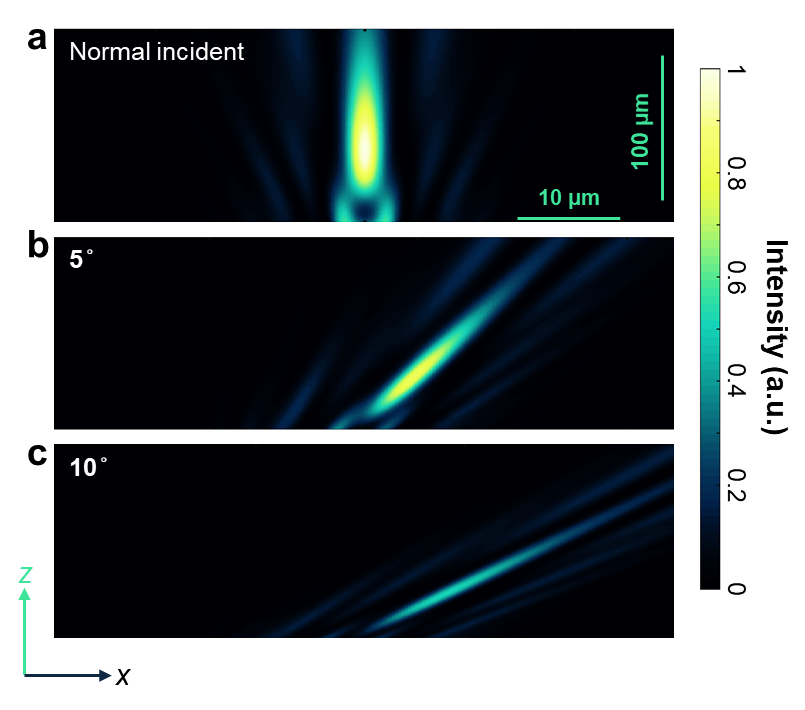
**

**Figure S16.** Simulated *x*–*z* intensity profiles of the metalens under (a) 0°, (b) 5°, and (c) 10° incidence. At 5°, the lens retains partial focusing with reduced intensity and sidelobes. At 10°, the focus is significantly degraded, indicating the angular sensitivity of the metasurface.

1. **Simulated light propagation at various wavelengths**

Simulated intensity distributions along the propagation direction (z) for wavelengths ranging from 525 nm to 550 nm using the finite-difference time-domain (FDTD) method. The metalens has a diameter of 14.4 μm and a numerical aperture (NA) of 0.15. The results illustrate the wavelength-dependent focal shift and intensity profile, revealing the chromatic behavior of the designed metalens under water immersion (**Figure S17**).

**
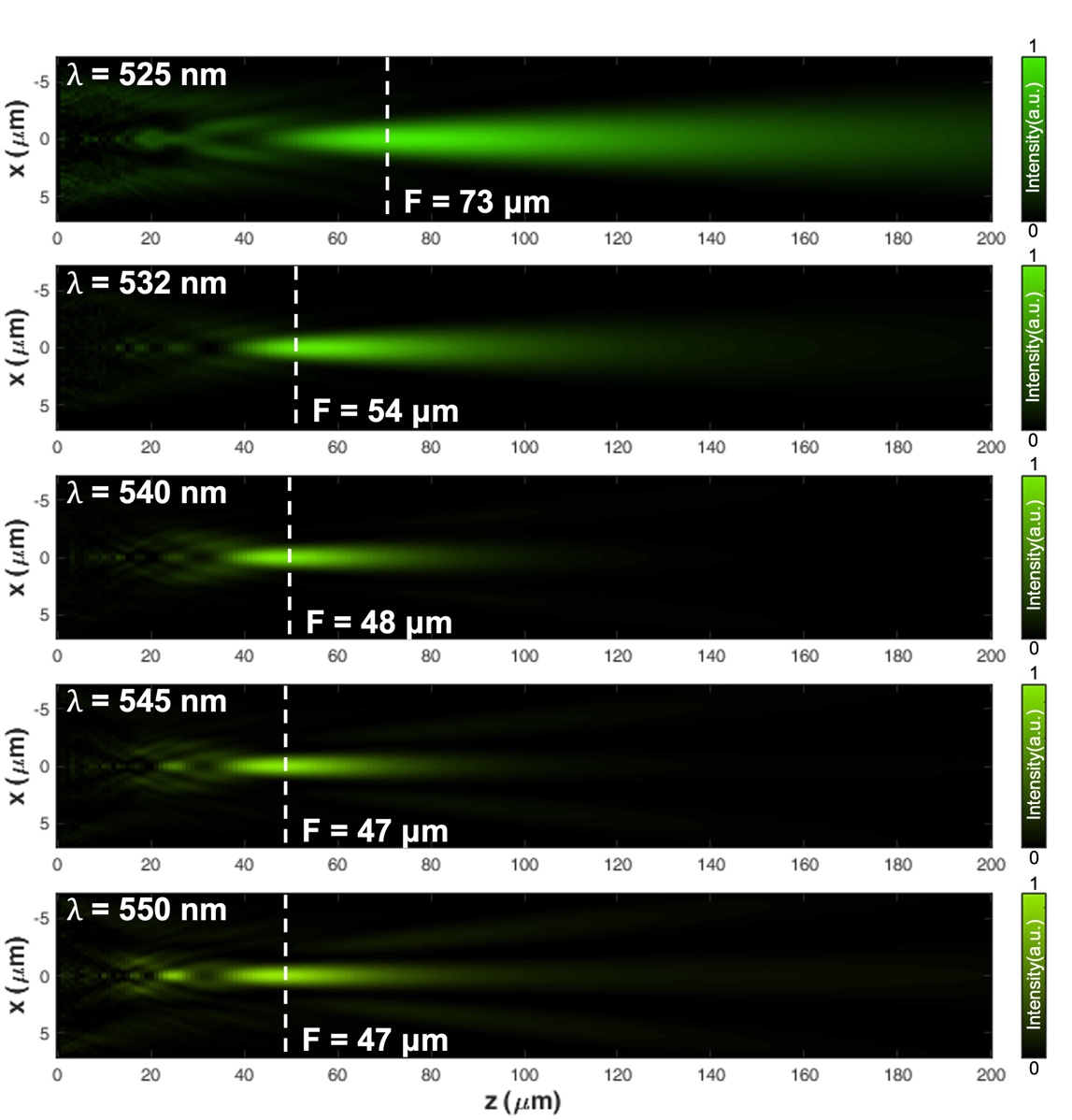
**

**Figure S17. Simulated light intensity profiles for the metalens with NA = 0.15 at various incident wavelengths.** The white dashed line indicates the position of the focal plane.

1. **Proximity effect calibration layouts for HIRUs**

The actual input patterns for electron beam lithography (EBL), are uniformly reduced in size due to process constraints. The proximity effect was corrected during the test of the fabrication process (**Figure S18**).


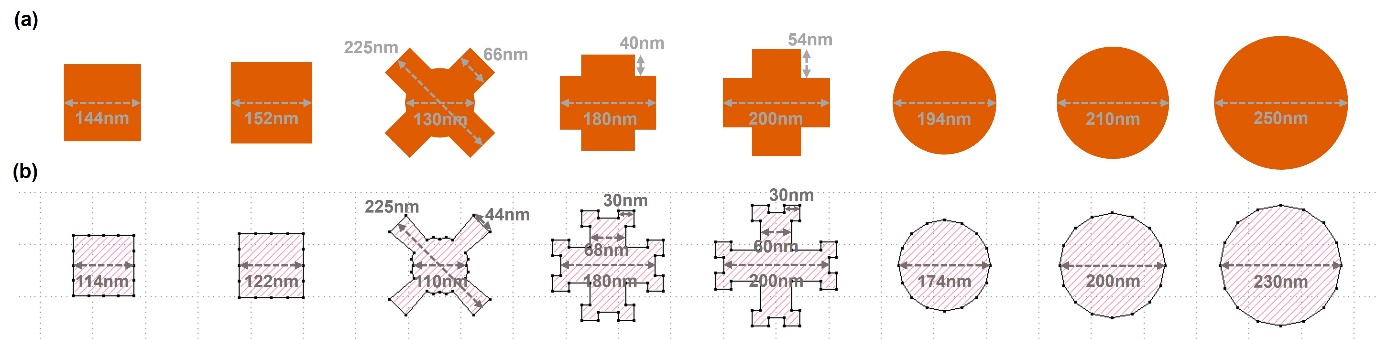


**Figure S18.** **Comparison of unit cell designs and the corresponding scaled-down input patterns for electron beam lithography (EBL).** (a) The originally designed structures include squares, crosses, and circles with various dimensions. (b) The actual input patterns for EBL.

**References for Supporting Information**

[1] T. Hinamoto, M. Fujii. "MENP: an open-source MATLAB implementation of multipole expansion for nanophotonics." *OSA Continuum* 4, no. 5 (2021): 1640.

[2] R. Alaee, C. Rockstuhl, I. Fernandez-Corbaton. "An electromagnetic multipole expansion beyond the long-wavelength approximation." *Optics Communications* 407 (2018): 17.

[3] M. Kerker, D. S. Wang, C. L. Giles. "Electromagnetic scattering by magnetic spheres." *Journal of the Optical Society of America* 73, no. 6 (1983): 765.

[4] A. Hassanfiroozi, Y. C. Cheng, S. H. Huang, et al. "Toroidal‐Assisted Generalized Huygens’ Sources for Highly Transmissive Plasmonic Metasurfaces." *Laser Photonics Reviews* 16, no. 6 (2022): 2100525.

[5] W. Liu, Y. S. Kivshar. "Generalized Kerker effects in nanophotonics and meta-optics [Invited]." *Optics Express* 26, no. 10 (2018): 13085.

[6] Y. C. Peng, Y. J. Wang, K. H. Chen, et al. "Deep-Ultraviolet AlN Metalens with Imaging and Ultrafast Laser Microfabrication Applications." *Nano Letters* 25, no. 8 (2025): 3141.

[7] Y. Shen, S. Pidishety, I. Nape, A. Dudley. "Self-healing of structured light: a review." *Journal of Optics* 24, no. 10 (2022).

[8] Y. Luo, M. L. Tseng, S. Vyas, et al. "Metasurface-Based Abrupt Autofocusing Beam for Biomedical Applications." *Small Methods* 6, no. 4 (2022): e2101228.

[9] H. T. Su, L. Y. Wang, C. Y. Hsu, et al. "Topology Optimization Enables High-Q Metasurface for Color Selectivity." *Nano Letters* 24, no. 33 (2024): 10055.

[10] R. Xu, J. Takahara. "Angle-insensitive Huygens’ metasurfaces of quadrupole modes." *Applied Physics Express* 15, no. 12 (2022): 122003.
